# Fosciclopirox suppresses growth of high-grade urothelial cancer by targeting the γ-secretase complex

**DOI:** 10.1038/s41419-021-03836-z

**Published:** 2021-05-31

**Authors:** Scott J. Weir, Prasad Dandawate, David Standing, Sangita Bhattacharyya, Prabhu Ramamoorthy, Parthasarathy Rangarajan, Robyn Wood, Amanda E. Brinker, Benjamin L. Woolbright, Mehmet Tanol, Tammy Ham, William McCulloch, Michael Dalton, Gregory A. Reed, Michael J. Baltezor, Roy A. Jensen, John A. Taylor, Shrikant Anant

**Affiliations:** 1 Department of Cancer Biology, University of Kansas Medical Center, Kansas City, KS USA; 2 Department of Pharmacology, Toxicology and Therapeutics, University of Kansas Medical Center, Kansas City, KS USA; 3 Institute for Advancing Medical Innovation, University of Kansas Medical Center, Kansas City, KS USA; 4 Department of Urology, University of Kansas Medical Center, Kansas City, KS USA; 5 Biotechnology Innovation and Optimization Center, University of Kansas, Lawrence, KS USA; 6 School of Pharmacy, Istanbul Kemerburgaz University, Istanbul, Turkey; 7 CicloMed LLC, Kansas City, MO USA; 8 Alba BioPharm Advisors Inc., Durham, NC USA; 9 The Gnomon Group, Carrboro, NC USA; 10 Department of Pathology and Laboratory Medicine, University of Kansas Medical Center, Kansas City, KS USA

**Keywords:** Bladder cancer, Pharmacodynamics

## Abstract

Ciclopirox (CPX) is an FDA-approved topical antifungal agent that has demonstrated preclinical anticancer activity in a number of solid and hematologic malignancies. Its clinical utility as an oral anticancer agent, however, is limited by poor oral bioavailability and gastrointestinal toxicity. Fosciclopirox, the phosphoryloxymethyl ester of CPX (Ciclopirox Prodrug, CPX-POM), selectively delivers the active metabolite, CPX, to the entire urinary tract following parenteral administration. We characterized the activity of CPX-POM and its major metabolites in in vitro and in vivo preclinical models of high-grade urothelial cancer. CPX inhibited cell proliferation, clonogenicity and spheroid formation, and increased cell cycle arrest at S and G0/G1 phases. Mechanistically, CPX suppressed activation of Notch signaling. Molecular modeling and cellular thermal shift assays demonstrated CPX binding to γ-secretase complex proteins Presenilin 1 and Nicastrin, which are essential for Notch activation. To establish in vivo preclinical proof of principle, we tested fosciclopirox in the validated N-butyl-N-(4-hydroxybutyl) nitrosamine (BBN) mouse bladder cancer model. Once-daily intraperitoneal administration of CPX-POM for four weeks at doses of 235 mg/kg and 470 mg/kg significantly decreased bladder weight, a surrogate for tumor volume, and resulted in a migration to lower stage tumors in CPX-POM treated animals. This was coupled with a reduction in the proliferation index. Additionally, there was a reduction in Presenilin 1 and Hes-1 expression in the bladder tissues of CPX-POM treated animals. Following the completion of the first-in-human Phase 1 trial (NCT03348514), the pharmacologic activity of fosciclopirox is currently being characterized in a Phase 1 expansion cohort study of muscle-invasive bladder cancer patients scheduled for cystectomy (NCT04608045) as well as a Phase 2 trial of newly diagnosed and recurrent urothelial cancer patients scheduled for transurethral resection of bladder tumors (NCT04525131).

## Introduction

Bladder cancer is the 6th most common solid tumor in the United States with an estimated 83,730 new cases and 17,200 deaths in 2021^1^. There are an estimated 699,450 people living with this disease in the United States^2^. The disease has a high risk of recurrence and progression that requires life-long surveillance, making this the most expensive cancer to treat on a per-patient-lifetime-basis^3^. While the overall 5-year survival rate for bladder cancer is 77%, in those with advanced disease, this rate can be as low as 4%^4^.

Bladder cancer is defined as two diseases, each with different treatment approaches and outcomes. Most newly diagnosed patients have non-muscle-invasive bladder cancer (NMIBC), which is generally considered less life-threatening than muscle-invasive bladder cancer (MIBC)^5^. However, in spite of endoscopic resection followed by intravesical immunotherapy (Bacillus Calmette-Guerin (BCG)) or chemotherapy (e.g., Mitomycin C, Gemcitabine), 60–70% of those with NMIBC will recur and 20–30% will progress to MIBC^6^, where the gold standard treatment is radical cystectomy coupled with cisplatin-based chemotherapy^7^. Additionally, patients who progress from NMIBC to MIBC have worse survival than those who present with de novo MIBC^8^.

Ciclopirox is a synthetic antifungal agent approved for topical dermatologic use in the treatment of a broad spectrum of fungal organisms^9^. The active pharmaceutical ingredient is either the free acid (CPX) or olamine salt (CPX-O). CPX is thought to act through the chelation of polyvalent metal cations, such as Fe^3+^ and Al^3+^, resulting in the inhibition of the metal-dependent enzyme systems (e.g., cytochromes, catalase, and peroxidase), and disruption of mitochondrial electron transport processes and energy production^10^. Preclinical studies have also shown anticancer activity of CPX in numerous solid tumors and hematologic cancers^11–25^. CPX decreased cell growth and viability of primary acute myeloid leukemia (AML) cells^11^. Moreover, CPX inhibited Wnt signaling^21^. Oral administration of CPX-O in patients with relapsed or refractory hematologic malignancies resulted in low circulating plasma CPX concentrations and Grade 3 dose-limiting gastrointestinal toxicities^26^. These observations led to the conclusion that CPX lacks clinical utility as an oral anticancer agent. Unfortunately, CPX and CPX-O also lack solubility properties necessary to formulate the drug as an injectable product.

**Fig. 1:**
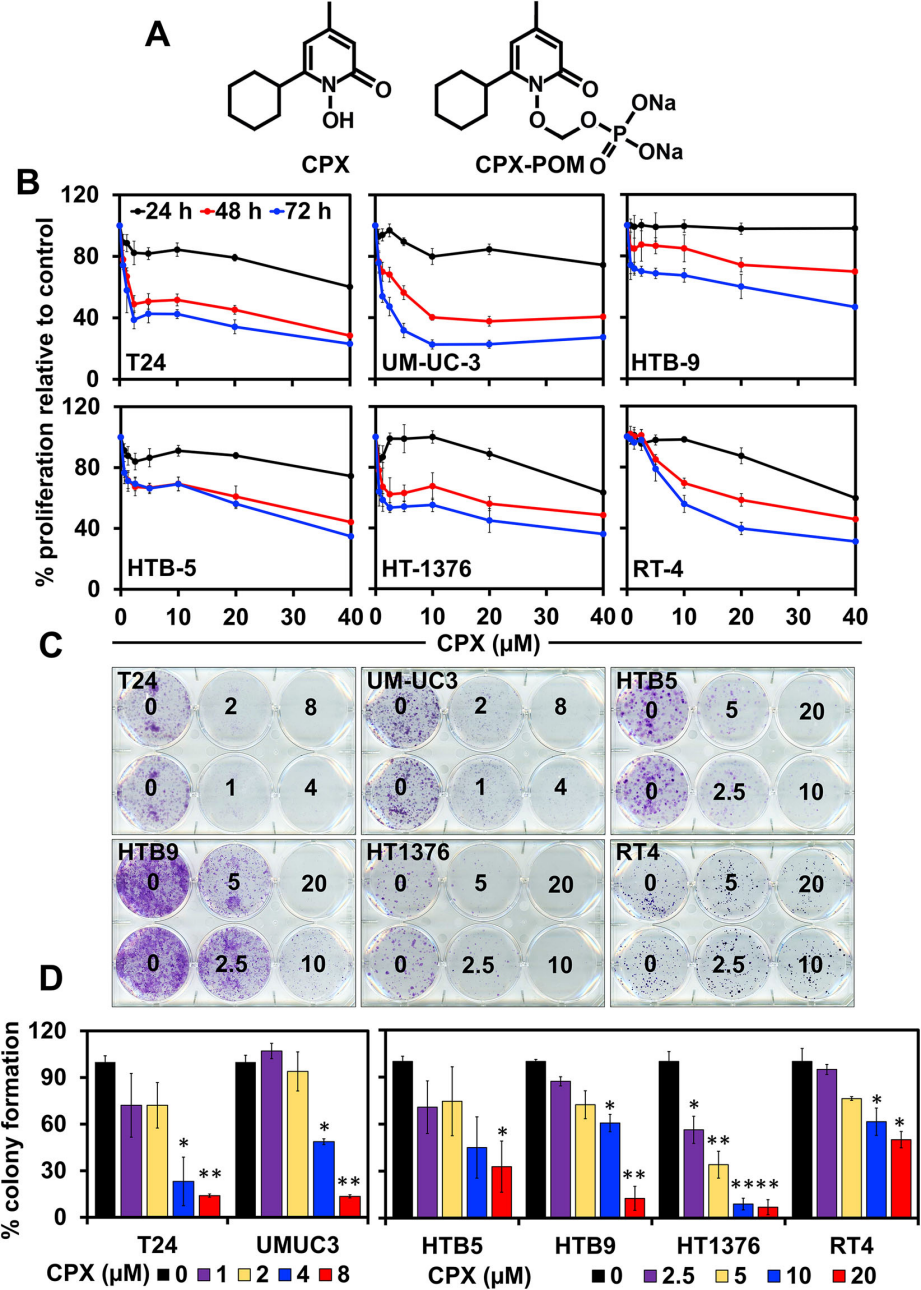
CPX inhibits proliferation and colony formation in bladder cancer cells. **A** Chemical structures of CPX and CPX-POM. **B** CPX inhibits proliferation of bladder cancer cell lines (T24, UM-UC-3, HTB-9, HTB-5, HT-1376, and RT-4). Cells were incubated with increasing concentration (0–40 μM) of CPX for up to 72 h. The treatment showed a significant dose and time-dependent decrease in cell proliferation when compared with untreated controls in the bladder cancer cell lines. **C** CPX inhibits colony formation. Cells were incubated with 0–20 μM CPX for 48 h and allowed to grow into colonies for 10 d. Incubation with CPX inhibits clonogenicity and number of colonies in bladder cancer cell lines (**D**).

In an effort to further evaluate CPX as an anticancer agent, we synthesized a phosphoryloxymethyl ester of CPX, fosciclopirox (Ciclopirox Prodrug, CPX-POM) (Fig. 1A). CPX-POM has outstanding water solubility and is readily formulated for parenteral administration. We then determined the activity of fosciclopirox in high-grade urothelial cancer in in vitro and in vivo preclinical models. We observed significant anticancer activity in several human high-grade bladder cancer cell lines. In addition, we observed activity in vivo employing a chemical carcinogen mouse model of bladder cancer. Mechanistically, we show that CPX binds to γ-secretase proteins Presenilin 1 and Nicastrin and inhibits Notch activation.

## Results

### Test article for in vitro studies

Following intravenous administration, CPX-POM is rapidly and completely metabolized to its active metabolite, ciclopirox (CPX) via circulating phosphatases^27^. CPX-POM is typically not detected in plasma nor urine in mice, rats, and dogs^27^. Once formed, CPX is extensively metabolized to form ciclopirox glucuronide (CPX-G) followed by urinary excretion^27^. The active metabolite is also excreted in urine, and as described herein, achieves steady-state urinary concentrations that exceed in vitro IC_50_ values by several fold at well-tolerated doses in mice. In vitro studies in the T24 high-grade human urothelial cancer cell line demonstrated that CPX-POM and CPX-G have little (IC_50_ > 50 µM) to no anticancer activity. As a result, in vitro studies described herein were conducted using the active metabolite, CPX, as the test article.

### CPX inhibits growth of bladder cancer cells

We first determined the effect of CPX on high-grade human urothelial cancer cell lines including T24, UM-UC-3, HTB-9, HTB-5, HT-1376, and RT-4 cells. Treatment with CPX significantly suppressed the proliferation of all six cell lines in a concentration- and time-dependent manner (Fig. 1B, Supplementary Fig. 1a). We next determined the long-term effect of short-term CPX exposure employing the clonogenicity assay. Treatment with CPX significantly suppressed the number of colonies in T24, UM-UC-3, HTB-9, HTB-5, HT-1376, and RT-4 cells (Fig. 1C, D). Furthermore, CPX exposure to an immortalized normal human urothelial cell line (UROtsa) demonstrated cytotoxicity at exposures exceeding 20 µM (Supplementary Fig. 1b), compared to IC_50_ values of 1.5–10 µM in the six urothelial cancer cell lines studied.

### CPX induces cell cycle arrest and apoptosis

**Fig. 2:**
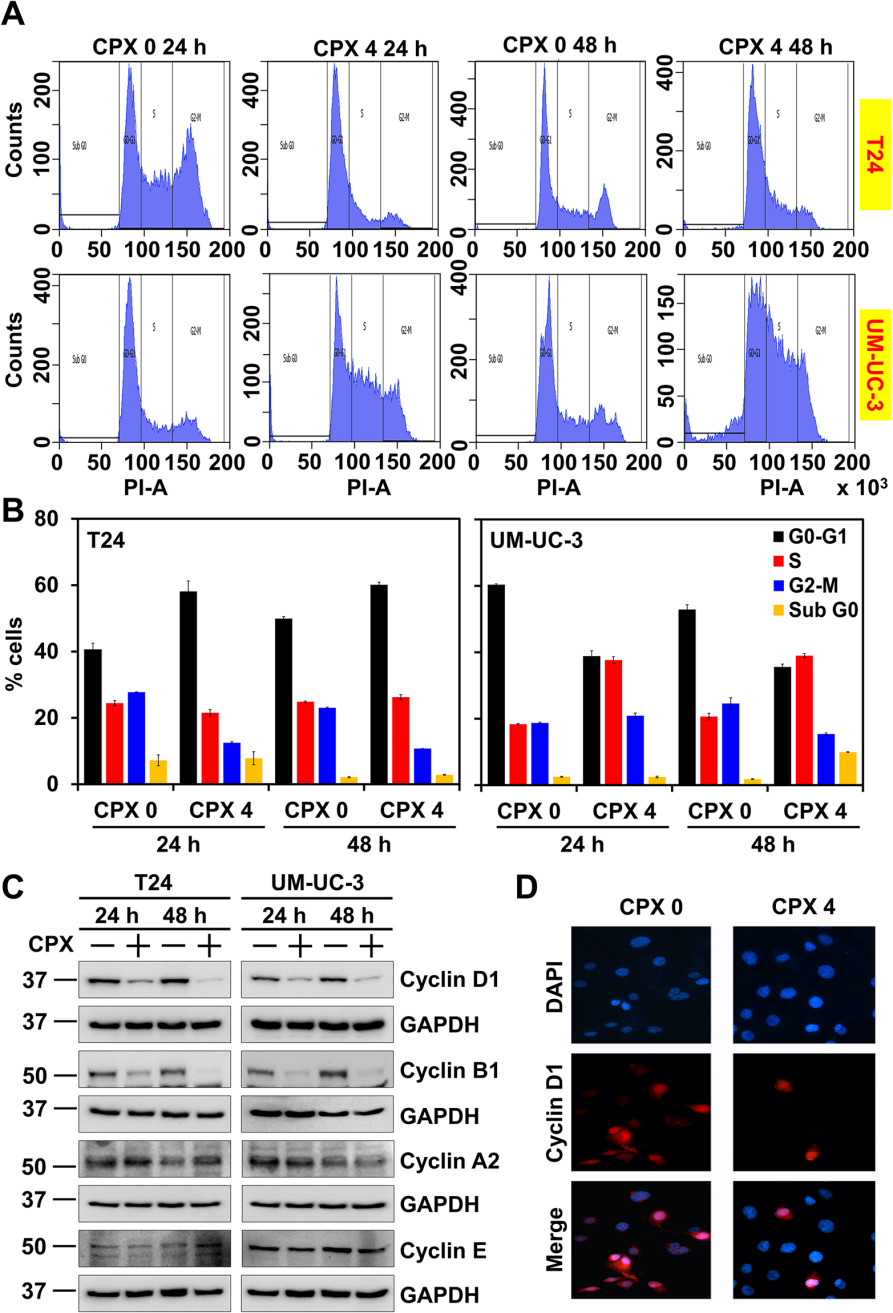
CPX induces cell cycle arrest in bladder cancer cell lines. **A** Cell cycle analysis of CPX-treated cells. T24 and UM-UC-3 cells were treated with 4 µM concentrations of CPX, and examined by flow cytometry. CPX treatment-induced G0/G1 arrest in T24 cells and S-phase arrest in UM-UC-3 cells. The percent cells in each phase are presented graphically (**B**). **C** CPX downregulates cyclin D1 and B1 expression in T24 and UM-UC-3 cells as assessed by western blot. **D** Immunofluroscence analysis shows downregulation of cyclin D1 expression after CPX treatment in T24 cells.

We next determined whether CPX mediated suppression of cell growth was due to effects on cell cycle progression. We treated T24 and UM-UC-3 cells with 4 µM concentrations of CPX followed by flow cytometric, analyses. Treatment with CPX resulted in a significant increase in cells in G0/G1 in T24 cells. In UM-UC-3 cells, a significant increase in cells in the S-phase of the cell cycle was observed. Together, these data suggest CPX induces cell cycle arrest (Fig. 2A, B).

**Fig. 3:**
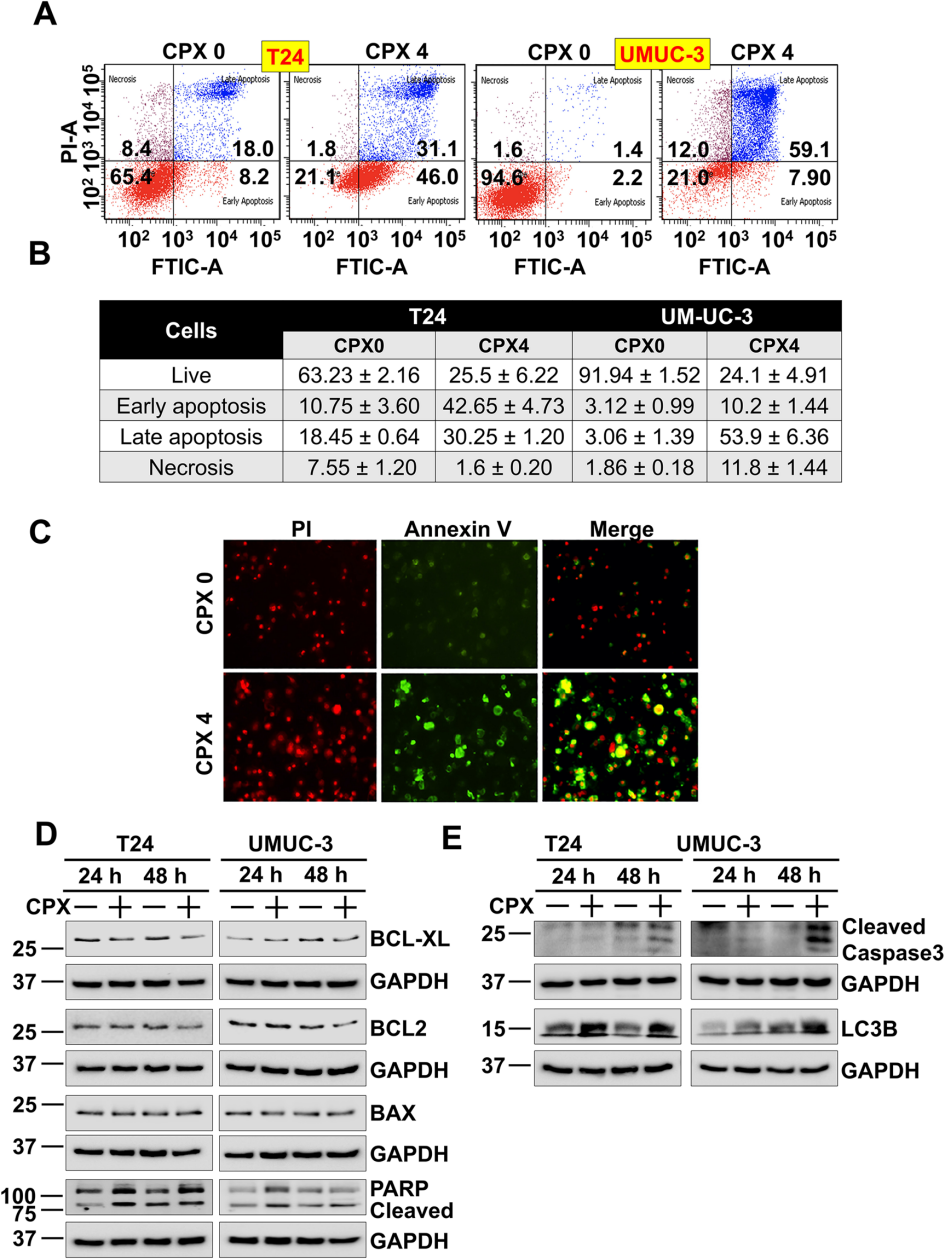
CPX induces apoptosis in bladder cancer cell lines. **A** T24 and UM-UC-3 cells were treated with CPX stained with Annexin V (FITC) and PI, and analyzed by flow cytometry. CPX treatment induced significant early and late apoptosis in T24 and UM-UC-3 cells. **B** The flow cytometric quantification of early and late apoptotic cells after CPX treatment over a period of 72 h in T24 and UM-UC-3 cells in Annexin-PI assay. **C** CPX induces apoptosis in T24 cells. T24 cells treated with CPX (4 µM) for 48 h were stained with Annexin V-FITC/PI solution and studied using immunofluorescence. CPX induced apoptosis in T24 cells. **D** Lysates from T24 and UM-UC-3 cells treated with CPX were studied by western blot for evaluating the effect on proteins involved in apoptosis. CPX treatment reduced the levels of antiapoptotic marker proteins Bcl2 and Bcl-XL. CPX treatment also increases the PARP cleavage compared to untreated controls. **E** CPX induces autophagy early which then followed by apoptosis. Lysates from T24 and UM-UC-3 cell treated with CPX (4 µM) for 24–48 h were analyzed by western blot. CPX treatment increased the expression of LC3B at 24 h and cleaved caspase-3 expression at 48 h. The data suggest that at early time points, CPX induces autophagy, which switches to apoptosis in T24 and UM-UC-3 cells.

Cyclins play a significant role in cell cycle progression. They bind to cyclin-dependent kinases and regulate different cell cycle transitions, and their deregulation can lead to tumor progression^28^. Therefore, we next determined the effect of 4 µM CPX on the expression of cyclin D1, B1, A2, and E in T24 and UM-UC-3 cells. Protein levels of Cyclin D1 and B1 were significantly reduced following CPX incubation at 4 µM in both T24 and UM-UC-3 cells (Fig. 2C). Given cyclin D1 plays a significant role in the progression from G1 to S-phase of the cell cycle, we also confirmed by immunofluorescence its expression was reduced significantly (Fig. 2D). Further, we determined whether CPX induced apoptosis utilizing Annexin V/PI staining and flow cytometry. We observed that when T24 cells were treated with CPX, there was an increase in the percentage of cells in the early and late apoptotic stage compared to untreated cells. In CPX-treated UM-UC-3 cells, an increase in the percentage of cells in the late apoptotic stage was also detected compared to untreated cells (Fig. 3A-C). There was a time-dependent increase in the number of early and late apoptotic cells over 72 h post-treatment. We confirmed apoptosis by immunofluorescence for annexin V, a critical protein that is expressed when cells are undergoing apoptosis (Fig. 3D). Exposure to CPX reduced levels of the antiapoptotic marker proteins Bcl-xL and Bcl-2 in both T24 and UM-UC-3 cells (Fig. 3E). In addition, there were increases in the levels of cleaved PARP protein in these cell lines after CPX treatment (Fig. 3E). Cell death can result from multiple pathways, including autophagy and apoptosis. The key effector proteins for autophagy and apoptosis are LC3B^29^ and activated caspase-3^30^, respectively. We observed that 24 h following incubation with CPX at 4 µM, there was a significant increase in the generation of LC3B protein levels in T24 and UM-UC-3 cells, while at 48 h, we observed increases in both LC3B and cleaved caspase-3 (Fig. 3F). Taken together, these data suggest that at early time points, CPX induces urothelial cancer cells to undergo autophagy which subsequently switches to apoptosis.

### CPX inhibits bladder cancer spheroid growth and cell migration

**Fig. 4:**
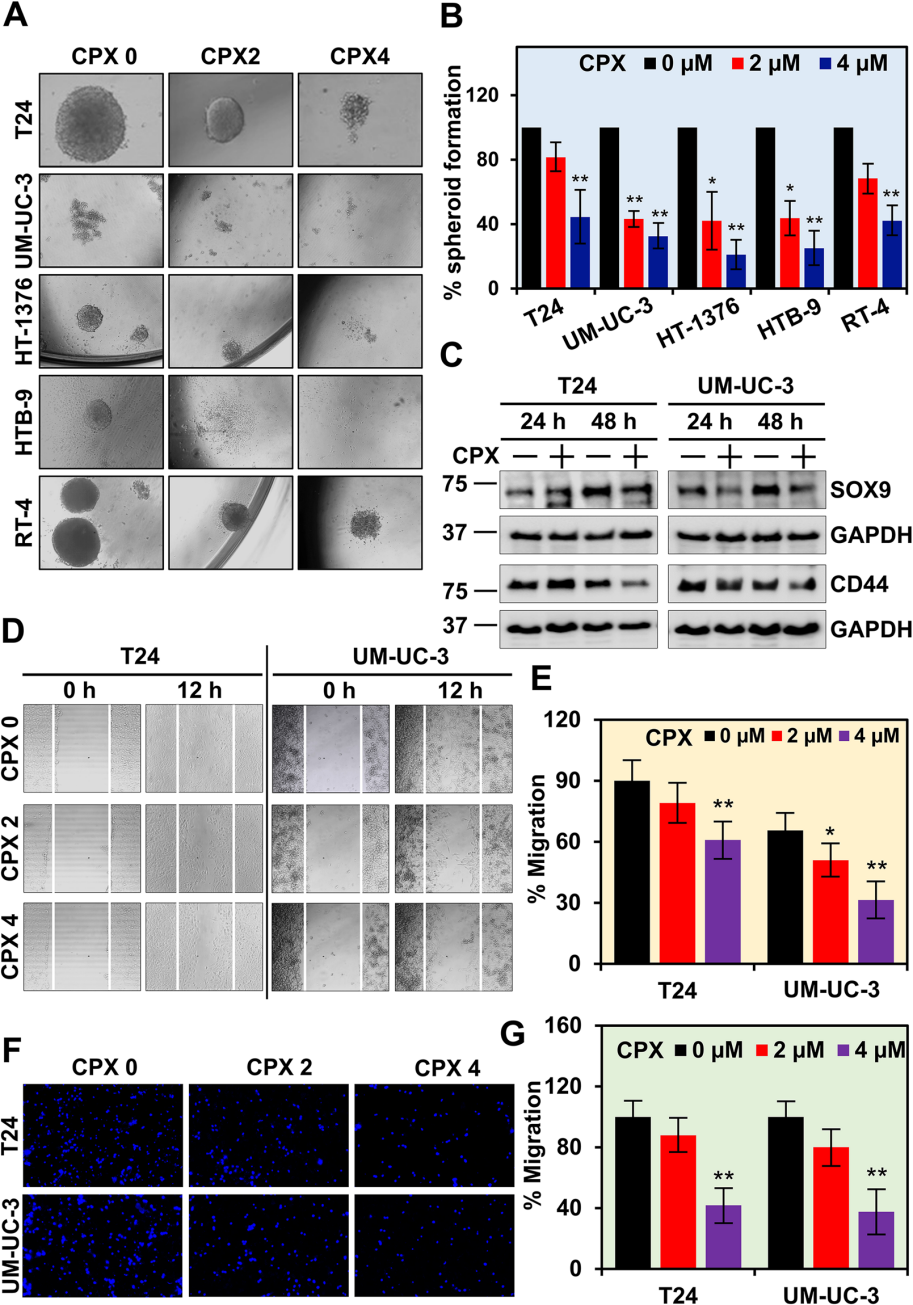
CPX inhibits bladder cancer spheroid growth and cell migration. **A, B** CPX inhibits spheroid growth. CPX suppressed the size (**A**) and number (**B**) of spheroids of bladder cancer cell lines. Cells were grown in spheroid growth media in ultralow adherent plates and treated with CPX 2 µM and 4 µM concentrations for 5 days. CPX significantly decreased bladdosphere formation in the T24, UM-UC-3, HT-1376, HTB-9, and RT-4 cell lines. **C** CPX inhibits the expression of bladder cancer stem cell marker proteins. Lysates from T24 and UM-UC-3 cell lines after CPX treatment were subjected to western blot analysis. CPX treatment inhibited the expression of SOX9 and CD44 in both cell lines. **D** CPX inhibits migration of T24 and UM-UC-3 cells. Both cell lines were grown to 95–100% confluency and a scratch was made to study the cell movement over a period of 12 h. CPX treatment reduced cell migration. **E** CPX inhibited the percent migration in both cell lines. **F** CPX inhibits migration of T24 and UM-UC-3 cells. CPX treatment significantly reduced the migration of both cell lines through transwell cell culture inserts (Boyden’s chamber). **G** The percent migraition is represented in graphical format.

A critical aspect of tumor survival is the presence of cancer stem cells (CSCs), which are believed to preserve themselves and divide asymmetrically to generate progenitors that can differentiate to form various cells in the tumor^31^. We determined whether CPX can also affect CSCs, by plating T24, UM-UC-3, HT-1376, HTB-9, and RT-4 cells in ultralow binding plates under conditions that favor the growth of spheroids. In the presence of CPX, there was a dose-dependent reduction in the size and number of primary spheroids (Fig. 4A, B). A critical experiment to confirm that cancer stem cells are affected is to take the primary spheroids exposed to CPX, and plate them in secondary and tertiary spheroids with no additional treatment. Replating of the primary spheroids exposed to CPX demonstrated that no spheroids developed, suggesting that CPX reduced the viability of CSCs. Furthermore, CPX treatment reduced the expression of bladder cancer stem cell marker proteins SOX9 and CD44 (Fig. 4C).

To analyze whether CPX further affects cell migration, we first performed the wound-healing assay, where scratch streaks were made on 90–95% confluent T24 and UM-UC-3 cells. After 24 h, closure of the cell-free areas was significantly reduced in the presence of CPX (Fig. 4D, E). These data indicated that CPX decreased the migratory ability of the cells. Further, we performed the Boyden chamber assay to characterize the effects of CPX on migration. We treated T24 and UM-UC-3 cells with CPX followed by measurement of cell migration over 12 h. CPX treatment significantly reduced the migration of bladder cancer cell lines (Fig. 4F, G). We also determined whether CPX inhibits cell invasion. For this, we plated T24 cells in Matrigel containing tissue culture inserts and after 24 h, cell invasion into the matrigel was determined. At the ½ IC_50_ of 2 µM, CPX significantly suppressed the invasion of T24 cells through the matrigel (Supplementary Fig. 2). These data suggest CPX suppresses bladder cancer migration and invasion, critical factors in cancer cell metastasis.

### CPX targets Notch signaling by inactivating γ-secretase proteins

**Fig. 5:**
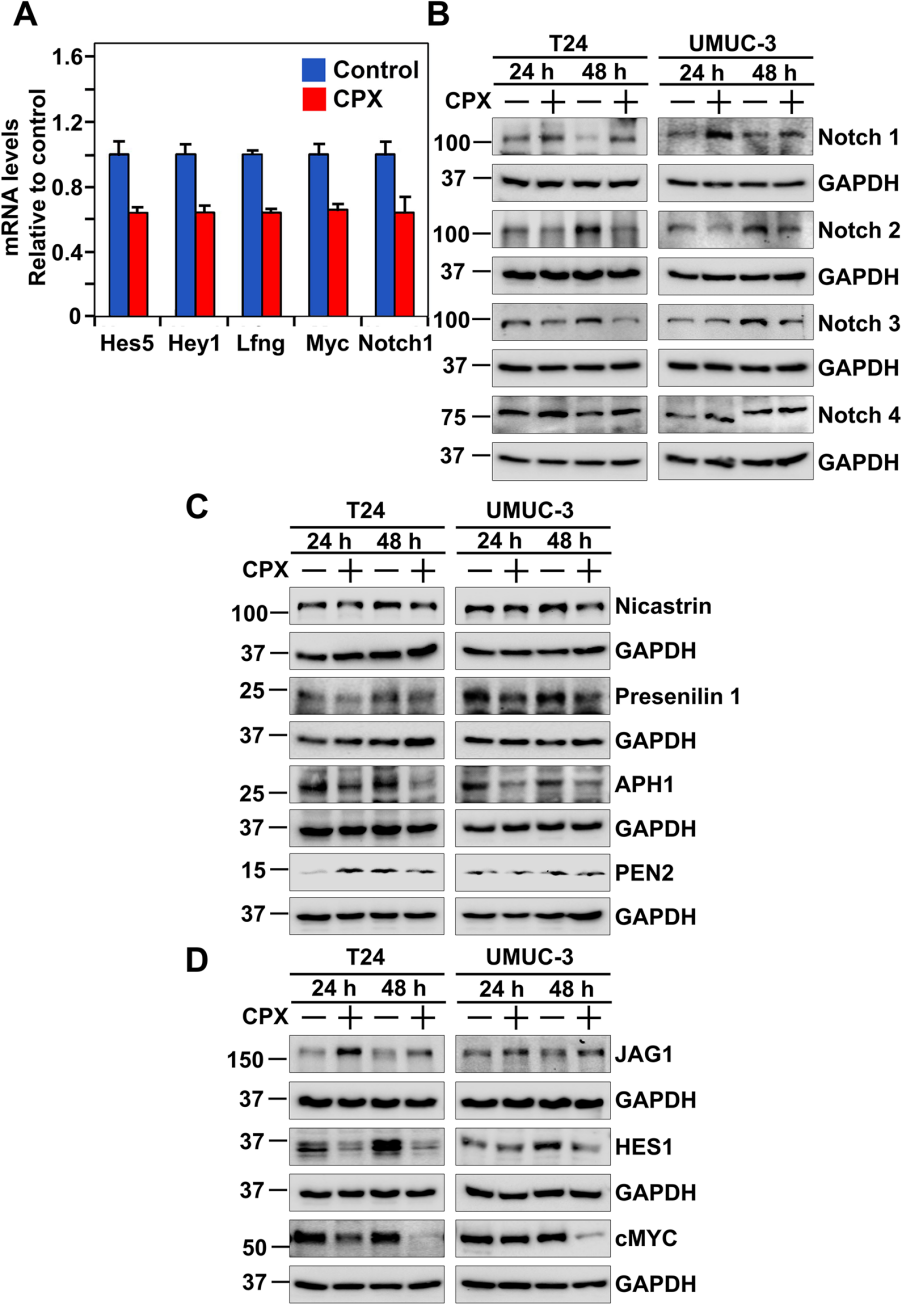
CPX targets the Notch signaling pathway, through inhibition of the γ-secretase complex. **A** CPX inhibits mRNA expression of genes involved in Wnt, Hedgehog, and Notch cell signaling pathways. RNA extracted from T24 cells treated with and without CPX for 48 h was used to generate cDNA. The samples were analyzed using a Human stem cell RT2 profiler PCR array to identify signaling pathways that important for stem cell maintenance like Notch signaling and Wnt signaling. Expression of Hes5, Lfng, Myc, and Notch 1 mRNAs were significantly downregulated after CPX treatment. **B** CPX affects the expression of Notch receptors. Western blot analysis demonstrates that CPX treatment increased the expression of Notch 1 and suppressed the activation of Notch 2 and Notch 3, while CPX has little to no effect on Notch 4. **C** CPX suppresses γ-secretase pathway proteins. Western blot analysis of CPX-treated T24 and UM-UC-3 cell lysates showed CPX treatment reduced levels of Nicastrin, Presenilin 1, APH-1, and PEN-2. **D** CPX suppresses notch signaling pathway ligand and downstream proteins. CPX increased the expression of ligand Jagged 1 and inhibited the expression of downstream targets Hes1, cMYC, and Cyclin D1 in T24 and UM-UC-3 cells.

Several published studies report that CPX targets ribonucleotide reductase to suppress cell transition from G1 to S-phase of the cell cycle^11^. Ribonucleotide reductase is an iron-dependent enzyme that is essential for DNA synthesis, and iron chelators such as deferoxamine inhibit their activity in proliferating cells^32,33^. To determine whether CPX inhibits urothelial cancer cell proliferation by primarily chelating iron, we tested whether deferoxamine also affects urothelial cancer growth. Deferoxamine affected neither T24 cell proliferation nor spheroid formation (Supplementary Fig. 3a, b). With the suggestion that inhibition of urothelial cancer cell proliferation is mediated by mechanism(s) other than, or in addition to inhibition of ribonucleotide reductase, we performed a qPCR-based array to examine the expression of proteins from various pathways following CPX treatment. We chose a stem cell-based array and observed inhibition in the expression of genes at the mRNA level involved in the Notch signaling pathway in T24 cells treated with 4 µM CPX (Fig. 5A). The Notch pathway is reported to possess tumor-promoting and tumor-suppressor roles in cancer^34,35^. It has been suggested that Notch signaling represents an attractive target for the treatment of bladder cancer. Notch is a transmembrane receptor that is activated by the serial cleavage activities of ADAM/TACE and γ-secretase proteins. These cleavage events result in the release of the intracellular domains (NICD) that can translocate to the nucleus to activate the expression of its target genes. There are four Notch receptor proteins^36^, and western blot analysis demonstrates that CPX treatment to T24 and UM-UC-3 cells enhanced the expression of Notch 1, suppressed the expression of Notch 2 and Notch 3, with no effect on Notch 4 expression (Fig. 5B). Next, we evaluated the effect of CPX on γ-secretase complex proteins in T24 and UM-UC-3 cells. CPX treatment inhibited the expression of Presenilin 1, Nicastrin, APH-1, and PEN-2 in both cell lines (Fig. 5C). Moreover, exposure to 4 µM CPX increased the expression of ligand Jagged 1 while decreasing the expression of downstream target proteins Hes1, cMyc, and Cyclin D1 in both T24 and UM-UC-3 cell lines (Fig. 5D).

### The CPX-POM maximum tolerated dose (MTD) is 470 mg/kg

We determined the maximum tolerated IP once-daily dose of CPX-POM to support dose selection for the first in vivo bladder cancer preclinical proof of principle study. We administered acute (single) IP doses of fosciclopirox ranging from 470, 700, and 930 mg/kg to three cohorts of mice. The acute IP MTD was defined as 470 mg/kg for CPX-POM. Subsequently, ¼ acute MTD (117.5 mg/kg), ½ acute MTD (235 mg/kg), and acute MTD (470 mg/kg) doses of CPX-POM were then administered once-daily IP for ten consecutive days to three cohorts of healthy mice. No drug-related clinical observations were observed in any treatment group, confirming 470 mg/kg CPX-POM as the subchronic MTD in mice (data not shown).

The completed fosciclopirox First-in-Human Dose Escalation Phase 1 (NCT03348514) trial in advanced solid tumor patients defined the MTD in humans as 900 mg/m^2^ administered intravenously over 10 min^37^. Based on the safety, dose tolerance, pharmacokinetics and pharmacodynamics of CPX-POM in advanced solid tumor patients, the MTD of 900 mg/m^2^ infused intravenously over 20 min was selected as the Recommended Phase 2 Dose (RP2D). The ongoing fosciclopirox Phase 1 expansion cohort study in muscle-invasive bladder cancer patients scheduled for cystectomy (NCT04608045) as well as ongoing Phase 2 trial in newly diagnosed and recurrent urothelial cancer patients scheduled for transurethral resection of bladder tumors (NCT04525131) are evaluating the RP2D. The human MTD (and RP2D) is quite comparable to the subchronic MTD established in mice of 470 mg/kg (which is equivalent, based on body surface area, to 1343 mg/m^2^ in the mouse).

### CPX-POM selectively delivers CPX to the entire urinary tract

**Fig. 6:**
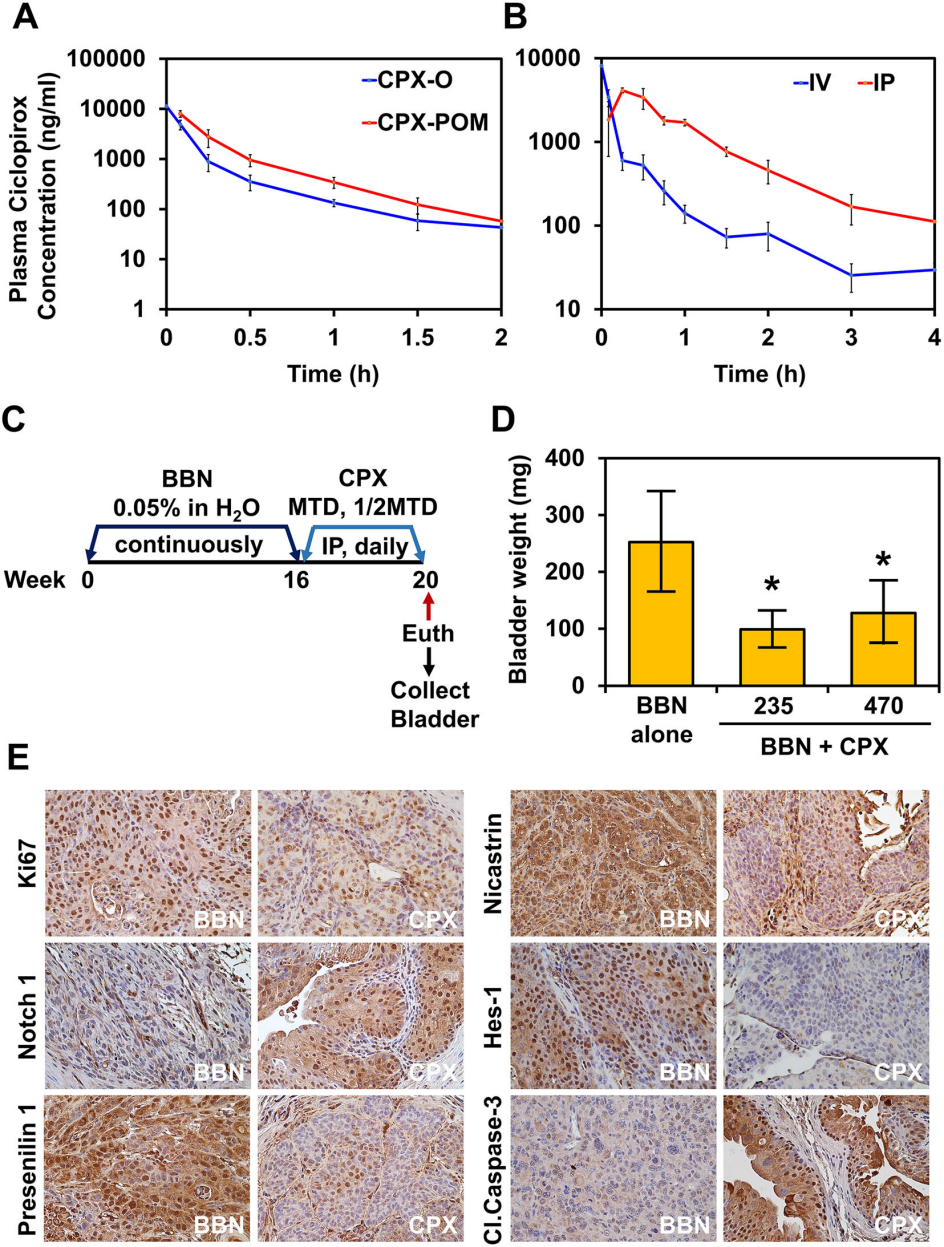
CPX-POM treatment suppresses bladder tumorigenesis in vivo in the validated BBN mouse model of bladder cancer. **A** Mean (±SD) plasma ciclopirox (CPX) concentration-time profiles following IV administration of 13.4 mg/kg CPX-POM and 18.1 mg/kg CPX-O to three C57BL/6 mice per serial blood collection time point per treatment group demonstrating rapid and complete metabolism of CPX-POM to its active metabolite, CPX. **B** Mean (±SD) plasma CPX concentration-time profiles following administration of 23.4 mg/kg IV and 117.5 mg/kg IP CPX-POM to three C57BL/6 mice per serial blood collection time point per treatment group demonstrating acceptable bioavailability of CPX following IP administration of CPX-POM. **C** Male C57BL/6 mice received 0.05% of N-butyl-N-(4-hydroxybutyl)-nitrosamine (BBN) in the drinking water for 16 weeks. Mice were treated with vehicle, 235 mg/kg CPX-POM (½MTD), or 470 mg/kg (MTD) CPX-POM IP once daily for 4 weeks (weeks 17–20). After 20 weeks, mice were euthanized, bladder was collected weighed, and subjected to further analysis. **D** CPX-POM treatment significantly reduced the bladder weight as compared to the vehicle-treated cohort at well-tolerated doses (*p* < 0.05). There were no statistically significant differences in bladder weights between the 235 mg/kg (½ MTD) and 470 mg/kg (MTD) CPX-POM treatment cohorts. **E** Immunohistochemistry analysis of bladder tumors obtained from CPX-POM-treated mice show a lower number of PCNA-positive nuclei compared to bladder tumors obtained from vehicle-treated mice. Bladder tumors obtained from CPX-POM treated mice also showed reduced expression of Notch 1, Presenilin 1, Hes1, and Nicastrin and increased levels of cleaved caspase-3 compared bladder tumors obtained from vehicle-treated mice.

The rate and extent of metabolism of CPX-POM was determined in mice receiving CPX-POM and CPX-O at 10 mg/kg IV. As illustrated in Fig. 6A, systemic CPX exposure as measured by area under the plasma CPX concentration-time curve, was similar following administration of the olamine salt of CPX compared to CPX-POM demonstrating complete metabolism of CPX-POM to CPX (data not shown). Complete metabolism of CPX-POM to CPX was also observed in rats and dogs^27^. Given this study demonstrated pharmacokinetic proof of principle, we then determined the absolute bioavailability of CPX-POM following IP administration (compared to the IV route of administration) to support in vivo preclinical proof of principle studies in the validated BBN mouse model of bladder cancer. The rate of absorption of CPX following IP administration of CPX-POM was rapid with maximal plasma CPX concentrations observed at 15 min post-dose (Fig. 6B). Mean (±SD) plasma CPX concentrations declined rapidly at a similar rate following both routes of administration. CPX-POM was not detectable in plasma in any blood (plasma) sample collected following IV or IP administration, demonstrating rapid and complete metabolism of the prodrug to its active metabolite. Plasma CPX pharmacokinetic parameters derived from the resultant plasma CPX concentration-time data are summarized in Supplementary Table 1. The absolute bioavailability of IP CPX-POM in mice was 60% as measured by systemic exposure to the active metabolite. Significant (10 µM) systemic CPX exposure was achieved following IP administration of 117.5 mg/kg CPX-POM (¼ MTD) with a mean C_max_ value of 4127 ng/mL. The apparent elimination half-life of CPX following both routes of administration was ~1 h. Systemic clearance of CPX was 7353 mL/h/kg. The extent of CPX distribution was moderate with a steady-state volume of distribution of ~5 L/kg.

In addition to characterizing systemic CPX exposure following IP administration of CPX-POM, urinary excretion of the active metabolite was characterized to describe exposure to the urothelium in mice. Single-dose and steady-state urinary excretion of CPX-POM, CPX, and CPX-G was characterized in mice following once-daily IP doses of 117.5 (¼ MTD), 235 (½ MTD), and 470 mg/kg (MTD) CPX-POM for ten consecutive days (Supplementary Fig. 4). Consistent with prior pharmacokinetic studies conducted in mice, CPX-POM was not detected in urine over the dose range studied, further demonstrating complete metabolism of the prodrug to the active metabolite. Despite the relatively short apparent elimination half-life of CPX, mean urine CPX concentrations following single and repeated IP CPX-POM doses were maintained over 24 h at concentrations well above the in vitro IC50 value of 4 µM. This study demonstrated that IP administration of CPX-POM at well-tolerated doses in mice, in addition to achieving adequate systemic exposure of CPX, delivers significant concentrations of the active metabolite topically to the urothelium. As a result, CPX-POM was evaluated in in vivo preclinical proof of principle studies.

### CPX-POM suppresses bladder tumorigenesis in vivo

To determine the effect of CPX-POM on bladder tumorigenesis, we used a chemical carcinogen-induced model that reproducibly establishes carcinoma in situ, and if not treated, progresses to MIBC^38^. Mice were given 0.05% BBN in drinking water ad libitum for 16 weeks (Fig. 6C). Subsequently, CPX-POM was administered at 235 (½ MTD) and 470 (MTD) mg/kg IP once daily for 4 weeks. Vehicle control-treated mice exposed to the chemical carcinogen demonstrated larger bladder weights than control animals not exposed to BBN. Bladder weights, a surrogate for tumor volume, in CPX-POM treated animals exposed to BBN were significantly (*p* < 0.05) less compared to vehicle control-treated mice exposed to the chemical carcinogen (Fig. 6D). Pathologic analysis revealed a distinct stage migration to lower stage tumors in the two CPX-POM-treated groups compared to vehicle control-treated mice (Supplementary Fig. 5). There was a moderate to a strong correlation between treatment and tumor stage (r^2^ = 0.71, *p* = 0.12). IHC for Ki67, as a marker of proliferation, showed high levels of positive staining in bladder tumor tissues obtained from mice exposed to just BBN. Following treatment with CPX-POM, however, there was a reduction in Ki67 staining and an increase in cleaved caspase-3 staining (Fig. 6E). Furthermore, IHC analysis of bladder tumor tissues demonstrated decreased staining for Notch 1, Presenilin 1, Hes1 and Nicastrin (Fig. 6E) in mice treated with CPX-POM. In control mice, who were not exposed to BBN but were treated with ½ MTD and MTD CPX-POM, there was no evidence of toxicity to the urinary tract based on histopathologic evaluation of kidneys, bladder, and ureters (data not shown).

### CPX targets γ-secretase proteins Presenilin 1 and Nicastrin

**Fig. 7:**
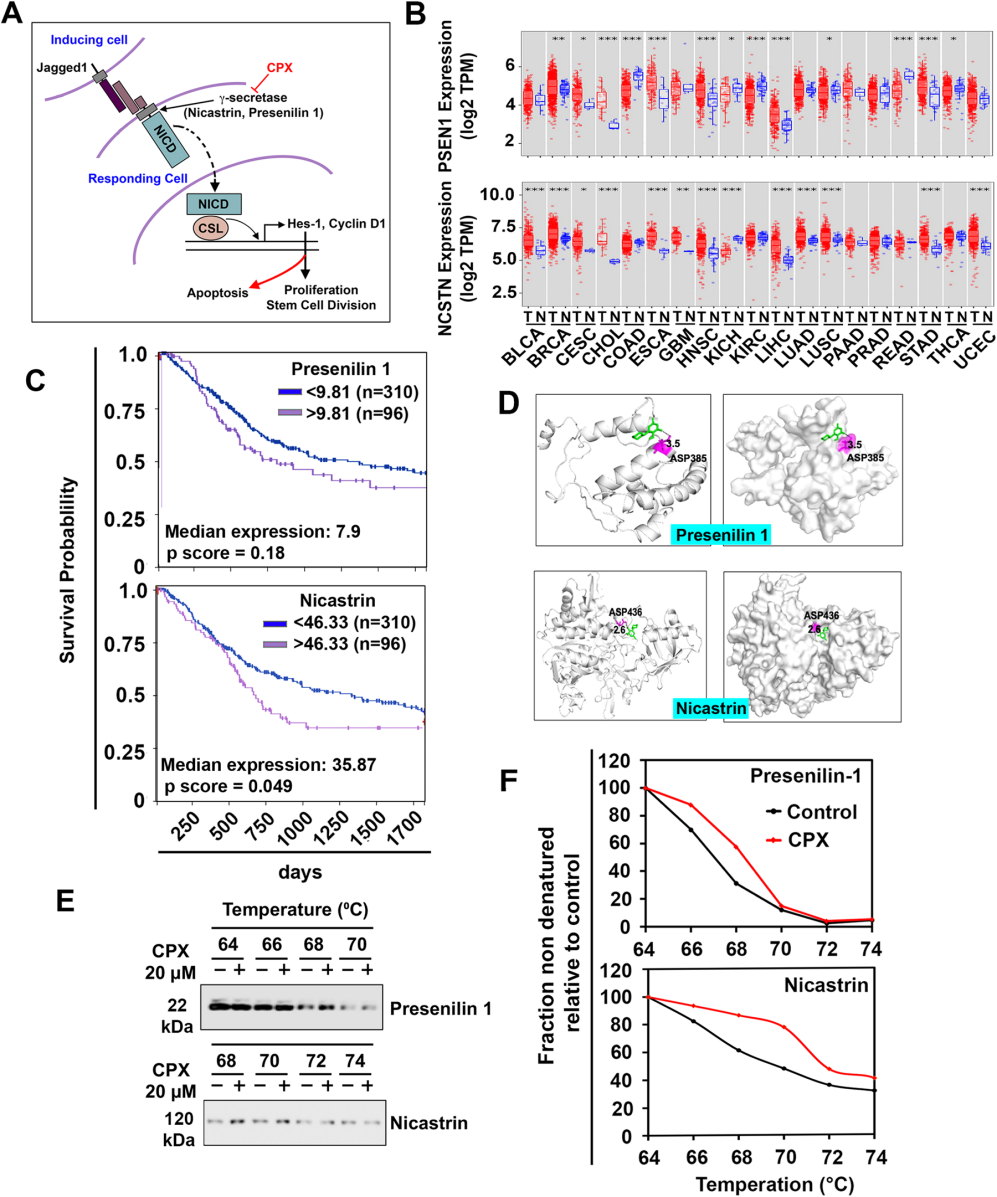
CPX targets γ-secretase complex proteins Presenilin 1 and Nicastrin. **A** Schematic representation showing the Notch signaling pathway. CPX targets γ-secretase target proteins Presenilin 1 and Nicastrin in bladder cancer cells. **B** mRNA expression of Presenilin 1 and Nicastrin in different cancer patients in the Cancer Genome Atlas database, extracted from Timer 2.0. (Bladder Urothelial Carcinoma [BLCA], Breast invasive carcinoma [BRCA], Cervical squamous cell carcinoma and endocervical adenocarcinoma [CESC], Colon Adenocarcinoma [COAD], Esophageal Squamous Cell Carcinoma [ESCA], Glioblastoma multiforme [GBM], Head and Neck squamous cell carcinoma [HNSC], Kidney renal clear cell carcinoma [KIRC], Liver hepatocellular carcinoma [LIHC], Lung adenocarcinoma [LUAD], Lung squamous cell carcinoma [LUSC], Pancreatic adenocarcinoma [PAAD], Prostate adenocarcinoma [PRAD], Rectum adenocarcinoma [READ], Stomach adenocarcinoma [STAD], Thyroid carcinoma [THEA], Uterine Corpus Endometrial Carcinoma [UCEC]). **C** Kaplan–Meier survival analysis showed that higher levels of Presenilin 1 or Nicastrin expression (*p* < 0.05) were significantly associated with poor overall survival in patients with bladder cancer. **D** The molecular docking analysis predicted the binding of CPX in the protein cavity of Presenilin 1 and Nicastrin. AutoDock Vina software program was used for molecular docking. **E** Cellular thermal shift assay (CETSA). T24 cell lysates were treated with CPX and subjected to differential temperature treatment for 3 mins and evaluated using western blot analyses. CPX protected Presenilin 1 and Nicastrin against thermal degradation, suggesting the active metabolite of CPX-POM interacts with these proteins. **F** Cumulative results from the densitometric evaluation of CETSA assay.

The data suggest that CPX downregulates the Notch pathway in bladder cancer cells by inhibiting the γ-secretase complex proteins Presenilin 1 and Nicastrin (Fig. 7A). We profiled the expression of Presenilin 1 and Nicastrin in 19 cancers within the Cancer Genome Atlas (TCGA) database using TIMER2.0 online web server, demonstrating that both genes had significantly higher expression in the several cancer types examined (Fig. 7B). Moreover, both the genes were upregulated in bladder cancer tissues compared with normal bladder tissue (Supplementary Fig. 6). Moreover, Kaplan–Meier survival analysis showed that higher levels of Presenilin 1 and Nicastrin expression (*p* < 0.05) were associated with significantly poorer overall survival in patients with bladder cancer (Fig. 7C). We next explored molecular docking using the Autodock Vina software program to gain insights into mechanisms of the inhibitory effects of CPX on the two proteins. The docking data predicted that CPX binds both Presenilin 1 and Nicastrin with the binding energy of −5.4 and −6.4 Kcal/mol, respectively (Fig. 7D). CPX stabilized itself by forming hydrogen bonds with Asp385 (3.5 Å) and Asp436 (2.6 Å) of Presenilin 1 and Nicastrin, respectively. The lower binding energy and hydrogen bonding of CPX to these proteins suggested the strong binding and a probable mechanism of inhibition of the γ-secretase complex. Moreover, CPX interacted with ASP385 residue of Presenilin 1, a residue that is essential for the catalytic activity of the protein. This region has been one of interest for designing γ-secretase complex inhibitors for the treatment of Alzheimer’s Disease and cancers^39^. Next, we used a cellular thermal shift assay (CETSA) to confirm the binding to the two proteins. T24 cell lysates were treated with vehicle or CPX for 4 h followed by denaturation using a thermal gradient. Thermal denaturation of Presenilin 1 and Nicastrin starts at 66 °C in vehicle-treated cells, while CPX treatment of T24 cells provided protection to thermal denaturation up to 68 °C and 70 °C for Presenilin 1 (Δ = 2 °C) and Nicastrin (Δ = 4 °C), respectively, (Fig. 7E, F). These data demonstrate that CPX inhibits Notch activation by binding directly to Presenilin 1 and Nicastrin.

## Discussion

NMIBC remains a significant problem with high rates of recurrence and progression to MIBC despite current standard-of-care therapy. Likewise, current treatments for MIBC and advanced disease rely on cisplatin-based chemotherapy. Approximately 50% of MIBC patients are either ineligible for cisplatin due to comorbidities, become resistant during treatment, or decline chemotherapy prior to cystectomy^40^. Although immunotherapy agents pembrolizumab and atezolizumab have been approved for the treatment of platinum-ineligible metastatic urothelial cancer patients whose tumors express high levels of PD-L1, response rates have only been around 25%^41^. Further, based on interim results from KEYNOTE-361 and IMVIGOR-130 trials demonstrating decreased survival, the FDA has warned against the use of these agents in untreated patients with low PD-L1 expressing tumors^42^. Thus, there is much room for improved outcomes in both NMIBC and MIBC.

Our studies demonstrate that in vitro exposure to the active metabolite of CPX-POM, ciclopirox, resulted in a significant dose- and time-dependent decrease in cell proliferation and colony formation of high-grade human urothelial cancer cell lines. Furthermore, CPX exposure significantly decreased spheroid formation and cancer stem cell marker proteins (CD44 and SOX9), suggesting that CPX acts, at least in part, through effects on CSCs. Further studies are ongoing to understand mechanisms by which CPX directly affects stemness, including determining the effect of the active metabolite on stem cell marker expression. Nevertheless, we have observed that CPX affects Notch signaling by targeting γ-secretase complex proteins Presenilin 1 and Nicastrin. Initial studies suggested a tumor-suppressor role for the Notch signaling pathway^43,44^. However, additional studies suggested that while the tumor-suppressor role may reside with Notch 1, Notch 2 acts more like an oncogene, and therefore, targeting Notch 2 may have merit^35,45^. Furthermore, Notch 3 was shown to be overexpressed in bladder cancer and is associated with poorer treatment outcomes^46^. Moreover, Notch 1 levels were found to be high in a population of cells marked by the putative stem cell marker ABCB2^47^. This suggests that while Notch 1 may not play a role in proliferation of bladder cancer cells, it has a key player in CSCs, resulting in aggressive tumor growth. Further studies are required to delineate the role of Notch proteins in cancer stemness, proliferation, migration, and invasion.

CPX exposure also significantly increased the percentage of cells arrested at S-phase and G0/G1, consistent with its ability to decrease cyclin D1. Initial studies have also suggested that CPX acts as a G1/S-phase transition blocker of a promyelocytic leukemia cell line HL60^22^. However, Ewing sarcoma cells treated with CPX were found to accumulate in the S-phase^13^. Moreover, this was observed as a result of inhibiting ribonucleotide reductase M2, the iron-dependent subunit of ribonucleotide reductase^11^. Initially, when CPX was found to inhibit urothelial cancer cell growth, we also explored if this was due to iron chelation. Deferoxamine, an iron chelator, did not affect urothelial cancer cell viability suggesting that the effects observed following CPX exposure are not due to suppressing ribonucleotide reductase activity^33^. On the other hand, we have observed that CPX binds γ-secretase complex proteins Presenilin 1 and Nicastrin to inhibit Notch signaling, suggesting that CPX targets the γ-secretase complex in bladder cancer cells. However, Notch receptors may not be the only substrate and that there may be additional targets. In this regard, proteomic profiling of HeLa cells for γ-secretase substrates identified multiple targets including the human leukocyte antigen, the low-density lipoprotein receptor and syndecan−1 and −2^38^. Nevertheless, these studies suggest that the effect of CPX may be dependent on the cell line.

Ciclopirox, as the free acid or olamine salt, lacks the water solubility necessary to formulate the drug as a parenteral drug product. In contrast, fosciclopirox (CPX-POM) possesses outstanding aqueous solubility and is easily formulated into an injectable solution. In vitro studies demonstrated that CPX-POM and CPX-G have little (IC_50_ > 50 µM) to no anticancer activity. Fosciclopirox is rapidly and completely metabolized to the active metabolite, CPX, via circulating, high capacity phosphatases^48^. As such, fosciclopirox meets the definition of a prodrug. IP administration of well-tolerated CPX-POM doses results in selective delivery of CPX to the entire urinary tract, not only achieving urine concentrations but also exceeding in vitro IC50 values by several fold. Urinary tract exposure to CPX at well-tolerated doses is associated with in vivo responses in the BBN model.

In contrast to standard-of-care NMIBC treatments administered to patients by bladder instillation (this route of delivery does not deliver drug to the upper urinary tract), systemic administration of CPX-POM delivers its active metabolite, CPX to the entire urinary tract, which is critical given the often-multifocal nature of urothelial carcinoma and its propensity for recurrence. To determine whether systemic and urinary tract drug exposure was sufficient to achieve in vivo antitumor response, we employed the BBN mouse model of bladder cancer rather than a xenograft model. As described above, this model reproducibly creates high-grade, basal-type tumors exclusively within the urinary tract without toxicity elsewhere in the body^49^. Without intervention, high-grade tumors progress to muscle-invasive disease. Based on our experiences evaluating several agents with different proposed mechanisms of action in the BBN model, we predefined criteria for drug response as ≥30% difference in bladder weights and ≥25% migration to lower stage tumors. We demonstrated a clear and selective effect on tumor development and tumor downstaging as a result of treatment with CPX-POM. In addition, we demonstrated inhibition of cell proliferation in bladder tumor tissues. There were also dose-dependent reductions in Notch 1, Presenilin 1, and Hes 1 in tissues from the BBN groups treated with CPX-POM supporting the hypothesis that CPX-POM acts, at least in part, by inhibiting Notch signaling. This BBN study demonstrated antitumor response at subchronic once-daily IP doses of 235 (½MTD) and 470 (MTD) mg/kg, thus, providing the rationale for advancing fosciclopirox to early phase clinical trials.

The safety, dose tolerance, pharmacokinetics and pharmacodynamics of CPX-POM has been characterized in 19 advanced solid tumor patients participating in the US multisite first-in-human Phase 1 trial (NCT03348514). Based on the safety, dose tolerance, pharmacokinetics, and pharmacodynamics of CPX-POM in advanced solid tumor patients, the MTD of 900 mg/m^2^ infused intravenously over 20 min was selected as the Recommended Phase 2 Dose (RP2D)^37^. A Phase 1 expansion cohort study is ongoing at three US sites, evaluating CPX-POM in cisplatin-ineligible and chemotherapy eligible muscle-invasive bladder cancer patients scheduled for cystectomy (NCT04608045). A Phase 2 trial in newly diagnosed and recurrent urothelial cancer patients scheduled for transurethral resection of bladder tumors is also ongoing at one US site (NCT04525131). The Phase 1 expansion cohort and Phase 2 CPX-POM trials are designed to establish clinical proof of concept, including demonstration of Notch signaling modulation in bladder tumors through γ-secretase complex inhibition. The fosciclopirox RP2D currently being studied in ongoing Phase 1 and Phase 2 clinical trials is quite comparable to the CPX-POM doses of 235 mg/kg (671.5 mg/m^2^) and 470 mg/kg (1343 mg/m^2^) demonstrating antitumor activity in the BBN mouse model.

## Methods and materials

### Test articles

CPX-POM is rapidly and completely metabolized in blood to its active metabolite, CPX, in mice, rats, and dogs via circulating phosphatases^27^. Given cell culture media lack phosphatases required to form CPX, ciclopirox olamine (CPX-O, Sigma–Aldrich, CAS Number 41621-49-2, molecular formula C_12_H_17_NO_2_, molecular weight 268.35) was purchased and used in the majority of in vitro studies (with the exception of in vitro studies conducted establishing that CPX-POM and the glucuronide metabolite of CPX possess minimal to no antiproliferative activity in urothelial cancer cells). Similarly, ciclopirox ß-D-glucuronide (CPX-G, Santa Cruz Chemical, CAS Number 79419-54-8, molecular formula C_18_H_25_NO_8_, molecular weight 393.39) was purchased to characterize in vitro activity of this metabolite. Ciclopirox Prodrug, disodium ((6-cyclohexyl-4-methyl-2-oxopyridin-1(2H)-yl)oxy) methyl phosphate heptahydrate (CPOX-POM, Fig. 1A), has a molecular formula of C_13_H_32_NNa_2_O_13_P and molecular weight of 487.35 g/mol. CPX-POM has been given the provisional International Nonproprietary Name (INN) of fosciclopirox. CPX-POM exists as a white solid, has excellent water solubility, and possesses solution stability for parenteral administration. For in vivo preclinical studies described herein, CPX-POM was supplied as a nonpreserved, sterile solution for IV and IP injection at a concentration of 50 mg/mL. The formulation included the active ingredient, CPX-POM disodium heptahydrate, sterile water for injection as a solvent, dibasic sodium phosphate (anhydrous) as a buffer, and sodium hydroxide and hydrochloric acid for pH adjustment.

### The cancer genome atlas (TCGA)

The gene expression and survival data of TCGA were analyzed by using the TIMER2.0 (http://timer.cistrome.org)^50,51^ and Human Protein Atlas (https://www.proteinatlas.org/).

### Cell lines

T24, UM-UC-3, HTB-5, HTB-9, RT-4, and HT-1376 cell lines were purchased from ATCC and used for this study. All cell lines were maintained as per ATCC’s media recommendations with 10% FBS and 1% penicillin/streptomycin. All experiments were performed before 10 passages.

### Cell proliferation assays

To determine the effect of CPX, the active metabolite of CPX-POM, on bladder cancer cell proliferation, T24, UM-UC-3, HTB-5, HTB-9, RT-4, and HT-1376 cells were exposed to several CPX exposure conditions during which concentration and exposure duration were varied. Five thousands cells per well were seeded on 96-well plates and grown overnight. Cells were then treated with decreasing concentrations of CPX (a range of 0–40 μM) in a Dulbecco’s modified Eagle’s Medium containing 10% fetal bovine serum. Cell proliferation by the hexosaminidase assay^52^ was measured at 24, 48, and 72 h following removal of CPX from cell culture. Briefly, the medium was removed and hexosaminidase substrate solution in citrate buffer pH 5 (7.5 mM), p-nitrophenol-N-acetyl-beta-D-glucosaminidase (Sigma–Aldrich) was added at 85 µL per well. The plate was incubated at 37 °C in 100% humidity for 30 min. The reaction was stopped with addition of 112.5 μL of 50 mM glycine containing 5 mM of ethylenediaminetetraacetic acid (pH 10.4). Absorbance was measured at 405 nm. We used four wells for each group and the experiment was repeated three times. Data were analyzed as a percent of control, where control wells were treated with equivalent amounts of dimethyl sulfoxide alone. For IC_50_ calculations, a plot between the drug concentration and hexosaminidase activity was generated and the data were fitted either linearly or exponentially. The best fit was used for further processing of data. IC_50_ for each human urothelial cancer cell line was determined as the CPX concentration resulting in 50% of maximal cell death observed after 48 h of continuous exposure.

### Colony formation assay

The effect of CPX on colony formation was assessed using the clonogenicity assay, in which six-well plates were seeded with 500 viable bladder cancer cells (T24, UM-UC-3, HTB-5, HTB-9, RT-4, and HT-1376) and grown overnight. The cells were then incubated in the presence or absence of various concentrations (0–20 µM) of CPX for 48 h. The CPX-containing medium was removed, and cells were washed in phosphate-buffered saline (PBS) and incubated for an additional 10 days in a drug-free medium; and the experiment was repeated three times. The colonies obtained were washed with PBS and fixed in 10% formalin for 10 min at room temperature then washed with PBS followed by staining with 1% crystal violet solution. The cell colonies formed were compared to those observed in untreated cells.

### Spheroid formation assay

Using the spheroid assay (bladdosphere assay) to examine the spheroid formation, bladder cancer cells were plated in ultralow binding plates. For the formation of spheroids, cells were cultured in DMEM supplemented with 20 ng/mL fibroblast growth factor-basic (Invitrogen), 10 mL per 500 mL of 50× B27 supplement (Invitrogen), epidermal growth factor 20 ng/mL (Invitrogen), and antibiotic and antimycotic solution. Cells were seeded at low densities (200–1000 cells/mL) in 96-well low-adhesion plates. Cells were treated with CPX at 1/2 IC50, and IC_50_ values generated using the cell proliferation assay described above. After 7 days of CPX incubation at these concentrations, control and bladder cancer spheroids were photographed. Four wells per group were used and the experiment was repeated three times.

### Scratch plate assay

Migration of bladder cancer cells was measured using an “in vitro wound-healing assay” (or scratch plate assay) performed in a six-well plate (Becton Dickinson). Briefly, T24 and UM-UC-3 cells were seeded and grown to near confluent monolayers in 10% FBS-supplemented culture medium. Using a sterile 10 μL pipette tip, perpendicular wounds were scratched through the cell monolayer followed by PBS washing two times. The scratched areas were photographed at ×10 magnification using computer-assisted microscopy. Cells were treated with vehicle and CPX at ½ IC_50_ and IC_50_ determined for this urothelial cancer cell line, and the experiment was repeated three times. After 12 h of incubation in a humidified incubator at 37 °C with 5% CO2, and each well was photographed at 10X magnification and analyzed for wound closure.

### Migration and invasion assay

Bladder cancer cell (T24 and UM-UC-3) migration and invasion were studied in a Boyden chamber consisting of a cell culture insert (8-μm pore polyethylene terephthalate membrane), seated in each well of a 24-well companion plate. T24 and UM-UC-3 cells were seeded in the upper chamber of an insert in serum-free media and positioned in a 24-well plate containing serum-containing media. The cells were treated with CPX at ½ IC_50_ and IC_50_. The migration is carried out in a humidified incubator at 37 °C with 5% CO_2_ for 12 h. After 12 h, inserts were removed, washed with PBS three times, and cleaned with cotton swabs to remove nonmigratory cells. The cells were fixed with 10% formalin, stained with DAPI and photographed and counted. For invasion assay, matrigel was thawed on ice and diluted in serum-free DMEM in a 1:1 ratio. 50 µL of Matrigel is added to a 24-well transwell insert and kept in a 37 °C incubator for 15–30 min to form a thin gel layer. T24 cells were plated on top of Matrigel in serum-free media and treated with CPX. Invasion was carried out in a humidified incubator at 37 °C with 5% CO_2_ for 12 h. Nonmigratory cells on the upper side of the insert containing Matrigel were removed with a cotton swab. Then, cells on matrigel were fixed with formalin and stained with Hoechst dye. For quantification, randomly selected fields on the lower side of each insert were photographed.

### Flow cytometry for cell cycle determination

Flow cytometry-based cell cycle analysis studies were conducted to determine the effect of CPX on cell cycle progression in T24 and UM-UC-3 bladder cancer cells. Cells were treated with 4 µM CPX, for 24 and 48 h. T24 and UM-UC-3 cells were then trypsinized and suspended in PBS. Single-cell suspensions were fixed using 70% ethanol for 2 h and subsequently permeabilized with PBS containing 1 mg/mL propidium iodide (Sigma–Aldrich), 0.1% Triton X-100 (Sigma–Aldrich), and 2 mg DNase-free RNase (Sigma–Aldrich) at room temperature. Flow cytometry was performed with a FACSCalibur analyzer (Becton Dickinson, Mountain, View, CA, USA), capturing 50,000 events for each sample. Results were analyzed with ModFit LT software (Verity Software House, Topsham, ME).

### Apoptosis assay

Immuno-fluorescent based studies were conducted to determine the effect of CPX on apoptosis in T24 and UM-UC-3 bladder cancer cells. Cells were seeded in six-well plates at 1 × 10^5^ cells/well and treated with 4 μM (IC_50_) CPX at 37 °C for 72 h. Next, T24 and UM-UC-3 cells were trypsinized and collected for the detection of apoptosis using Annexin V-FITC Apoptosis Detection Kit (eBioscience, San Diego, CA, USA). Briefly, T24 and UM-UC-3 cells were washed twice with cold PBS and resuspended in 500 µL binding buffer (10 mM HEPES-NaOH pH 7.4, 140 mM NaCl, 2.5 mM CaCl2) at a concentration of 1 × 10^6^ cells/ml. After the addition of 5 μl Annexin V-FITC solution and PI (1 μg/ml), the cells were incubated for 15 min at room temperature and then analyzed by Flow cytometry and Nikon Eclipse Ti microscope (Nikon Inc., Melville, NY).

### Quantitative PCR for cancer stem cell signaling pathway expression

Gene expression analysis was performed on T24 cells exposed to 4 µM CPX for up to 48 h using RT2 Profiler™ polymerase chain reaction (PCR) Arrays (SA Biosciences, Frederick, MD, USA) per the manufacturer’s instructions. The human signal transduction pathway finder PCR array was used (cat. no. PAHS-014ZA). Total RNA isolated from T24 cells using TRIZOL reagent was reverse transcribed with Superscript II reverse transcriptase in the presence of random hexanucleotide primers (all from Invitrogen, Carlsbad, CA). Complementary DNA (2 μg) was mixed with 1275 μL RT2 SYBR Green qPCR Master Mix (PA-010; SA Biosciences) to make up a total volume of 2550 μL. Each well received 25 μL of this mix. Quantitative PCR was performed on a Bio-Rad CFX machine following detection protocols recommended by SA Biosciences. Cycle threshold values for a reaction set were analyzed online at the manufacturer’s web site (http://www.sabiosciences.com/pcr/arrayanalysis.php). Changes in mRNA expression were expressed as fold change relative to control.

### Western blot analysis

Western blot analysis was used to determine the effect of CPX exposure on protein expression of key components of the Notch signaling pathway. T24 and UM-UC-3 cells were exposed to 4 μM CPX for 48 h, after which cells were collected and lysed with cell lysis buffer. Cell lysates collected following centrifugation were subjected to polyacrylamide gel electrophoresis and blotted onto Immobilon polyvinylidene difluoride membranes (Millipore, Bedford, MA, USA). Antibodies specific for the four Notch isoforms were purchased from Cell Signaling Technology (Beverly, MA, USA), Abcam Inc. (Cambridge, MA, USA) and Santa Cruz Biotechnology Inc. (Santa Cruz, CA, USA) and specific proteins were detected by the enhanced chemiluminescence system (GE Healthcare, Piscataway, NJ, USA). T24 and UM-UC-3 cells were exposed to 4 μM CPX for 48 h, after which time cells were collected and lysed with cell lysis buffer. Cell lysates collected following centrifugation were subjected to polyacrylamide gel electrophoresis and blotted onto Immobilon polyvinylidene difluoride (PVDF) membranes (Millipore, Bedford, MA, USA). The PVDF membranes were incubated overnight at 4 °C with primary antibodies specific for cyclin D1 (Cat no. 2978), B1 (Cat no.4138), A2 (Cat no.4656) and E (Cat no.4129), BCL2 (Cat no.15071), BCL-XL (Cat no. 2764), BAX (Cat no. 2772), PARP (Cat no.9532), cleaved Caspase-3 (Cat no.9664), LC3B (Cat no.2775), Notch 1–4 (Cat no.03608,5732, 5276, 2423), Jagged 1 (Cat no. 2155), Presenilin 1 (Cat no.5643), Nicastrin (Cat no.3632), APH-1 (Cat no. PA5-20317), SOX2 (Cat no. 3579), CD44 (Cat no. 37259,) and PEN-2 (Cat no.8598) were purchased from Cell Signaling Technology (Beverly, MA, USA) and used in 1:1000 dilution. GAPDH (Cat no. sc-365062) was purchased from Santa Cruz Biotechnology Inc. (Santa Cruz, CA, USA) and used in 1:2000 dilution. Next day, the membranes were washed and incubated with respective anti-mouse or anti-rabbit secondary antibody (Sigma–Aldrich, Saint Louis MO, USA) for 1 h at room temperature. The specific proteins were detected by the enhanced chemiluminescence system (GE Healthcare, Piscataway, NJ, USA). Western blots were visualized by Bio-Rad ChemiDoc-XRS+ instrument and analyzed by image lab software (Bio-Rad, CA, USA).

### Molecular docking

The AutoDock Vina software program^53^ was employed to study the interaction of CPX and γ-secretase complex proteins Presenelin-1 (PDB ID: 2KR6) and Nicastrin (PDB ID: 4R12)^54^. AutoDock Vina is freely available software which has shown high performance and accuracy for structural-based virtual screening of compounds. For docking analysis, the 3D-grid was generated surrounding the catalytic residue ASP385 in the case of Presenilin 1 and we used grid parameters as published by Pal et al.^55^ for Nicastrin. A grid center co-ordinate consisting of grid spacing of 1.0 Å and 40X40X40 point size was utilized. We used default parameters of the AutoDock Vina tools for preparation of proteins and CPX. Total Kollman and Gasteiger charges were added to proteins before performing docking and the Lamarckian generic algorithm was used to predict topmost conformations. About 10 conformations for each protein-CPX complex were studied. The most favorable conformation was selected based on the number of hydrogen bonds and the lowest binding energy. The resulting complex was evaluated and visualized using Pymol (https://pymol.org/2/)^56^.

### Cellular thermal shift assay (CETSA)

The ability of CPX to interact with Presenilin 1 and Nicastrin was evaluated by using CETSA^57^. Briefly, T24 cells were cultured and grown to 70–80% confluency. Cells were lysed using lysis buffer and diluted to the concentration of 3 mg/ml. The cell lysates were incubated with vehicle or CPX (20 μM) for 4 h. Next, the cell lysate was aliquoted into PCR tubes and subjected to heat gradient (44–80 °C) for 3 min. Subsequently, cells were centrifuged for 20 min. The resultant proteins were diluted with 4× Laemmli buffer, boiled at 95 °C for 5 min and loaded onto 10% SDS-PAGE gel, transferred to PVDF membrane and incubated overnight with Presenilin 1 (Cell Signaling # 5643) and Nicastrin (Cell Signaling # 3632) antibodies at a concentration of 1:1000. The western blot was visualized by Bio-Rad ChemiDoc-XRS+ instrument and analyzed by image lab software.

### Animals

Male C57BL/6 mice were selected for all in vivo preclinical studies consistent with the validated N-butyl-N-(4-hydroxybutyl)-nitrosamine (BBN) chemical carcinogen model of bladder cancer described below. The in-life portions of the maximum tolerated dose and pharmacokinetic studies were conducted at the AAALAC-accredited animal facility at Xenometrics LLC, Stilwell, KS. The BBN study was conducted at the University of Connecticut Health Center in Farmington, CT. All animal studies were approved by the Institutional Animal Care and Use Committees at the two institutions.

### Maximum tolerated dose study

The intraperitoneal (IP) acute and subchronic maximum tolerated doses (MTD) of CPX-POM were determined in fasted male C57BL/6 mice under study conditions according to the principles of Good Laboratory Practices (GLP). In the acute MTD phase, mice were given a single IP dose of CPX-POM. Doses were given under a flexible dosing regimen adopted from OECD Acute Oral Toxicity Test Guideline 425, Up-and-Down Acute Toxicity Procedure^58^. Doses were based on the tolerability, as determined by clinical observations, of the previous dose. Animals were observed for a minimum of two hours post-dose. Subsequent doses were adjusted based on observations of drug-related adverse effects (if any) from the previous dose. Dosing was staggered with five mice studied per dose group. Parameters examined included mortality, morbidity, clinical observations, and body weights. In dose cohort 1, IP doses were administered at a volume of 20 mL/kg resulting in a dose of 940 mg/kg as CPX-POM; in dose cohort 2, IP doses were administered at a volume of 10 mL/kg resulting in a dose of 470 mg/kg as CPX-POM; in dose cohort 3, IP doses were administered at a volume of 15 mL/kg resulting in a dose of 705 mg/kg as CPX-POM. Based on clinical observations from the first three treatment groups it was determined that additional dose groups were unnecessary. In the subchronic MTD phase, once-daily IP doses of CPX-POM were administered for 10 consecutive days to three treatment groups of ten mice each. Vehicle was administered IP once daily to a control group of ten mice as well. Dose levels for the subchronic MTD phase were selected based on the determination of the acute MTD. IP doses of test article were administered at volumes of 2.5 mL/kg, 5 mL/kg, and 10 mL/kg, resulting in doses of 117.5 mg/kg, 235 mg/kg, and 470 mg/kg, respectively, as CPX-POM. Doses were administered volumetrically based on the most recent body weight of the animal. Consistent with the acute MTD phase of this study, parameters examined included mortality/morbidity, clinical observations, and body weights. In addition, necropsies were performed on each animal with kidneys, liver, urinary bladder, and urinary tract tissues collected. Based on the absence of gross pathology findings, these tissues were not further processed.

### Pharmacokinetic studies

The pharmacokinetics of CPX-POM were characterized following IV and IP administration to 72 fasted male C57BL/6 mice. IP doses were administered at 117.5 mg/kg to Treatment Group 1 (*n* = 30) and 2 (*n* = 6), and IV doses at 23.5 mg/kg to Treatment Group 3 (*n* = 30) and 4 (*n* = 6). Serial blood (plasma) samples were collected via cardiac puncture from Treatment Groups 1 and 3 at the following time points: predose, 0.083, 0.167, 0.25, 0.5, 0.75, 1, 1.5, 2, 3, and 4 h post-dose. Metabolism Cages were used to collect complete urine from Treatment Groups 2 and 4 at the following urine collection intervals: predose, 0–4, 4–8, and 8–24 h post-dose.

Once the subchronic MTD of IP CPX-POM was defined, single-dose and steady-state urinary concentrations of CPX were characterized in six mice per dose cohort at MTD (470 mg/kg), ½ MTD (235 mg/kg), and ¼ MTD (117.5 mg/kg). Complete 24-h urine samples were collected using metabolism cages with three mice housed per cage per dose cohort. Urine samples were collected prior to dosing as well as over 0–8 h and 8–24-h collection intervals starting on days 1 (single dose) and 10 (steady-state). Urine samples were collected frozen on dry ice into glass collection jars, thawed, transfer into tared plastic jars, and weighed. All urine was stored at −20 ± 5 °C until shipment to the bioanalytical laboratory for analysis.

### In vivo preclinical proof of principle in a mouse model of bladder cancer

The N-butyl-N-(4-hydroxybutyl)-nitrosamine (BBN) chemical carcinogen model was selected to determine in vivo preclinical proof of principle. This is a well-known and validated model for bladder cancer^49^. It reproducibly creates carcinoma in situ at ~16 weeks of BBN exposure via the drinking water at 0.05% concentration. The tumors are genetically similar to the basal phenotype described in humans and is a suitable model for preclinical testing of novel agents^49^. Without intervention, carcinoma in situ observed following 16 weeks of exposure to BBN will progress from NMIBC to MIBC. Based on our experiences evaluating several agents with different proposed mechanisms of action in the BBN model, we predefined criteria for drug response as ≥30% difference in bladder weights and ≥25% migration to lower stage tumors. Seventy-five male C57BL/6 mice were divided into six treatment cohorts. Three cohorts of 19 animals each were exposed to BBN for 16 weeks, after which time mice were treated with either vehicle, 235 mg/kg CPX-POM, or 470 mg/kg CPX-POM IP once daily for 4 weeks (weeks 17–20). Three control treatment cohorts (six mice per group) received drinking water absent BBN. After 16 weeks, the three control cohorts were treated with either vehicle, 235 mg/kg CPX-POM, or 470 mg/kg CPX-POM IP once daily for 4 weeks (weeks 17–20). The vehicle contained 50 mM Captisol in 25 mM phosphate buffer, pH 7.0. Animals were inspected daily for general health, with weights recorded biweekly throughout the study. At the end of the study, the animals were euthanized. Bladders were harvested, weighed, then inflated with and placed in 10% buffered formalin in phosphate-buffered saline for 24 h then transferred to phosphate-buffered saline. Formalin-fixed bladders were bisected and embedded along the midsagittal plane and serially sectioned for whole-mount pathologic analysis. Evaluation of tissues was performed by a board-certified pathologist in a blinded fashion. Hematoxylin- and eosin-stained slides were assessed for tumor stage and grade. Immunohistochemistry for cell proliferation proteins Ki67 (Cat. No. AB9260, Sigma–Aldrich, St. Louis, MO, USA), apoptosis protein cleaved caspase-3 (Cat. No. 9579, Cell Signaling Technology, Beverly, MA, USA) were performed using UltraVision™ Quanto Detection System (ThermoFisher, Waltham, MA, USA). Immunohistochemistry for Notch signaling proteins was also performed, specifically the expression of Notch 1 (Cat. No.3608), Hes1 (Cat. No. 11988, Cell Signaling Technology, Beverly, MA, USA), Presenilin 1 (Cat.No. MA1-752), and Nicastrin (Cat. No. PA5-20304, ThermoFisher, Waltham, MA, USA).

### Bioanalysis

Mouse plasma and urine bioanalytical methods were developed and validated under the principles of GLP for three analytes including CPX-POM, the active metabolite CPX, and the inactive metabolite ciclopirox glucuronide (CPX-G). Methods employed liquid chromatography-tandem mass spectrometry (LC-MS/MS) to quantitate plasma and urine concentrations of CPX-POM metabolites. The upper (ULOQ) and lower limits of quantitation (LLOQ) for CPX-POM in plasma and urine were 100 and 5000 ng/mL, respectively. The ULOQ and LLOQ for CPX in both plasma and urine were 25 and 5000 ng/mL, respectively. The urine CPX-G method had ULOQ and LLOQ limits of 250 ng/mL and 5000 ng/mL.

### Pharmacokinetic data analysis

Nonparametric pharmacokinetic parameters were generated from resultant plasma and urine drug/metabolite concentration-time data using Phoenix WinNonlin (Certara™ Version 6.3) software. Nominal blood sampling times were used given blood (plasma) and urine samples were collected without exception per sampling times defined in study protocols. Predose plasma drug/metabolite concentrations reported as less than LLOQ were designated as below the quantifiable limit (BQL) and treated as zero in the calculation. BQL values observed in the terminal phase of the plasma drug/metabolite concentration-time profile were treated as missing in determination of the apparent first-order elimination rate constant and area under the plasma drug/metabolite concentration-time curve. Given the absence of quantifiable concentrations of CPX-POM in plasma and urine, pharmacokinetic data analysis was not performed for parent drug. Descriptive statistics (mean ± standard deviation) were calculated for the resultant plasma and urine CPX and CPX-G pharmacokinetic parameters. Inferential statistical analyses were not performed given the objectives of the two pharmacokinetic studies.

### Statistical analysis

All values are expressed as the mean ± SD. Data were analyzed using an unpaired two-tailed *t*-test. A *p*-value of less than 0.05 was considered statistically significant. Inferential statistical analyses were performed using SPSS software (IBM Corp., Somers, NY). In addition, a one-way analysis of variance (ANOVA) was performed for multiple comparisons. Post-hoc testing was performed with two-sided Dunnett’s tests. The number of tumors and tumor stage were compared across treatment groups using cross-tabulation and correlation with treatment evaluated with the Pearson Chi-Square test. *P*-values are represented as *p < 0.05, **p < 0.01, ***p < 0.001.

## Supplementary information

Legends_Supplementary Figures

Supplementary Figure 1

Supplementary Figure 2

Supplementary Figure 3

Supplementary Figure 4

Supplementary Figure 5

Supplementary Figure 6

Supplementary Table 1
