# Supplementary material for: Fosciclopirox suppresses growth of high-grade urothelial cancer by targeting the γ-secretase complex
Source: Cell Death Dis. 2021 May 31;12(6):562. doi: 10.1038/s41419-021-03836-z (PMC8166826; doi:10.1038/s41419-021-03836-z)
Supplement: Supplementary file 1 — Legends_Supplementary Figures [file 41419_2021_3836_MOESM1_ESM.docx]

**Supplementary Figure Legends**

**Supplementary Figure 1.** CPX inhibits proliferation of bladder cancer cell lines.

1. CPX inhibits proliferation of bladder cancer cell lines (T24, UMUC3, HTB-9, HTB-5, HT1376 and RT-4). Cells were incubated with increasing concentration (0–40 μM) of CPX for up to 72 h. The treatment showed a significant dose- and time-dependent decrease in cell proliferation when compared with untreated controls in the bladder cancer cell lines. IC_50_ values are summarized.
2. CPX did not affect the growth of immortalized normal urothelial cell line (UROTsa) at lower concentrations.

**Supplementary Figure 2.** CPX inhibits invasion of bladder cancer cell lines.

CPX inhibits invasion of T24 cells. T24 cells were plated in transwell cell culture inserts containing a layer of Matrigel and allowed to invade through Matrigel for 12 h. CPX treatment at 2 μM (1/2 IC50) and 4 μM (IC50) concentration significantly reduced the cell invasion.

**Supplementary Figure 3.** Deferoxamine does not affect T24 cells.

1. Cells were treated with increasing concentrations of Deferoxamine (0-10 µM) for up to 72 h. Data suggests CPX does not affect cell proliferation.
2. Cells were plated in ultra-low binding plated in stem cell growth media and allowed to develop bladdospheres. Treatment with 10µM deferoxamine did not affect bladdosphere formation.

**Supplementary Figure 4:** Average 24-hour urine CPX concentrations following single dose and repeat dose IP administration of 117.5, 235, and 470 mg/kg CPX-POM demonstrating systemic administration achieves steady-state urine concentrations of CPX that exceed *in vitro* IC_50_ values by several-fold. The subchronic maximum tolerated IP dose of CPX-POM is 470 mg/kg.

**Supplementary Figure 5.** Pathologic analysis revealed high-grade urothelial cancer with a distinct migration to lower tumor stages in the CPX-POM treated groups. There was moderate-to-strong correlation with treatment groups (r2= 0.71, p=0.12).

**Supplementary Figure 6:** Nicastrin and Presenilin 1 are upregulated in bladder cancer patients. Differential gene expression of Presenilin 1 and Nicastrin between normal tissues and tumor samples obtained from bladder cancer patients in the TCGA database obtained through Xena browser.

**Supplementary Table Legend**

**Supplementary Table 1.**  Plasma CPX pharmacokinetic parameters in C57BL/6 mice following single IV and IP doses of CPX-POM.
