# Supplementary figures and images for: Fosciclopirox suppresses growth of high-grade urothelial cancer by targeting the γ-secretase complex
Source: Cell Death Dis. 2021 May 31;12(6):562. doi: 10.1038/s41419-021-03836-z (PMC8166826; doi:10.1038/s41419-021-03836-z)

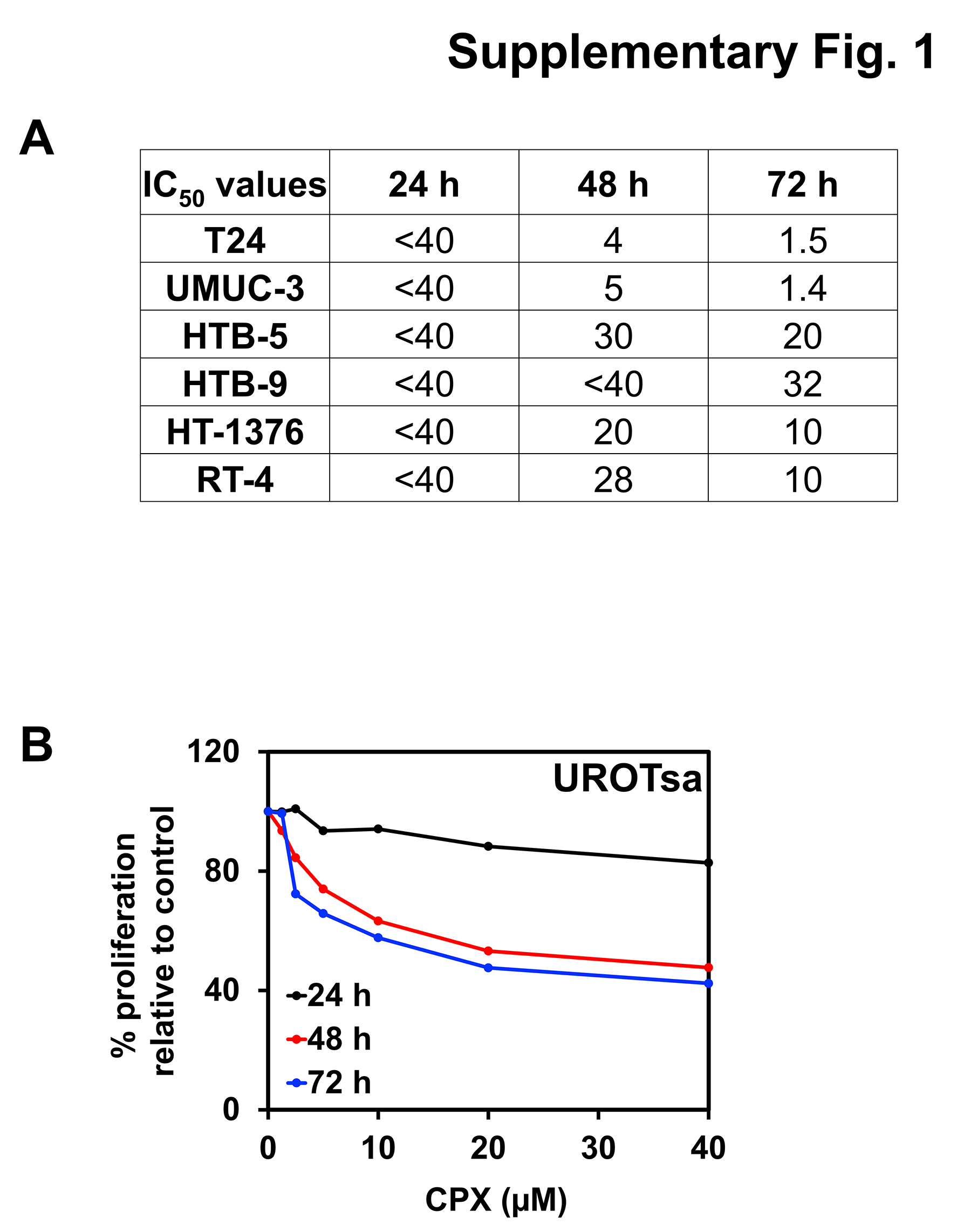

Supplement: Supplementary file 2 — Supplementary Figure 1 [file 41419_2021_3836_MOESM2_ESM.tif]

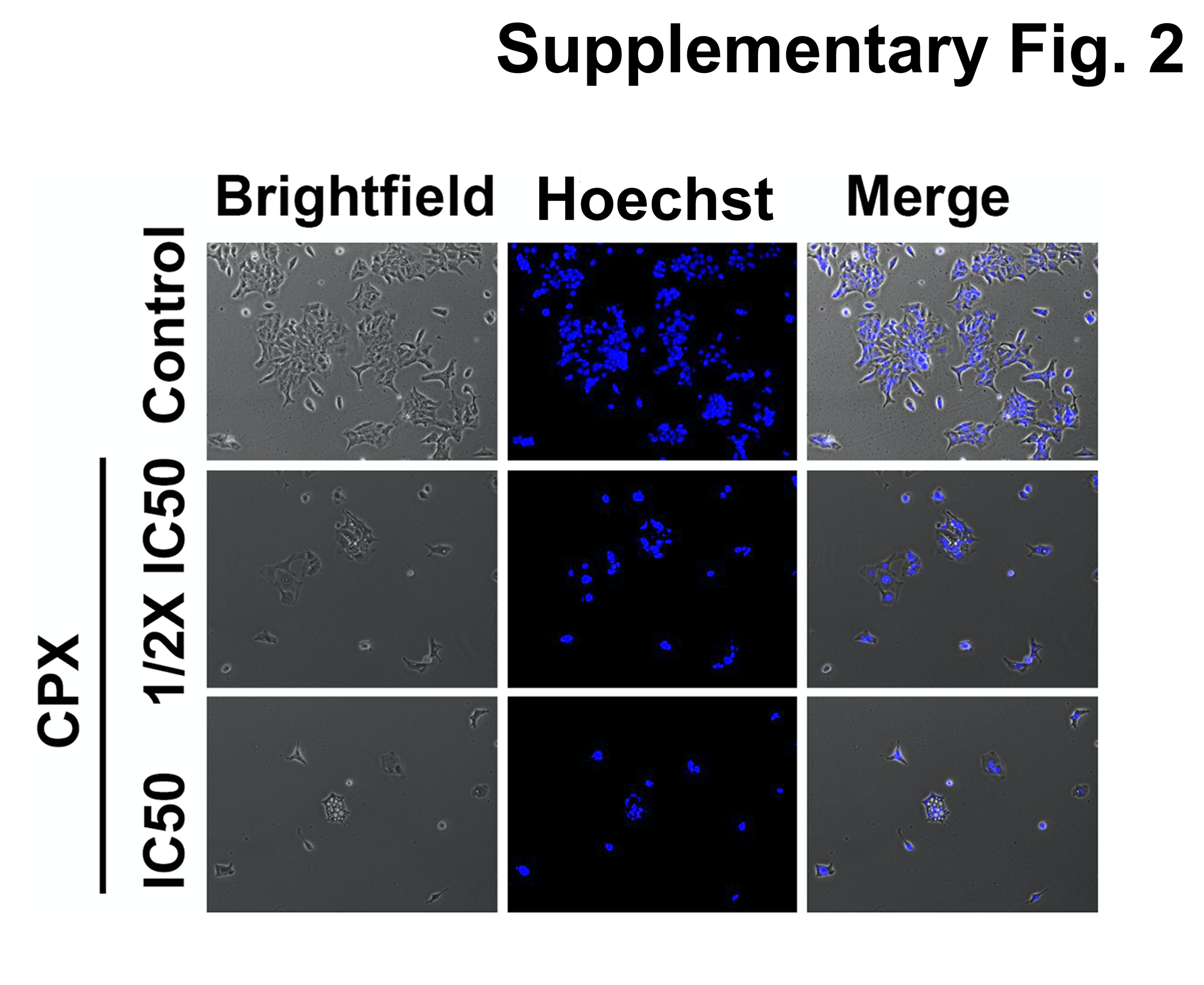

Supplement: Supplementary file 3 — Supplementary Figure 2 [file 41419_2021_3836_MOESM3_ESM.tif]

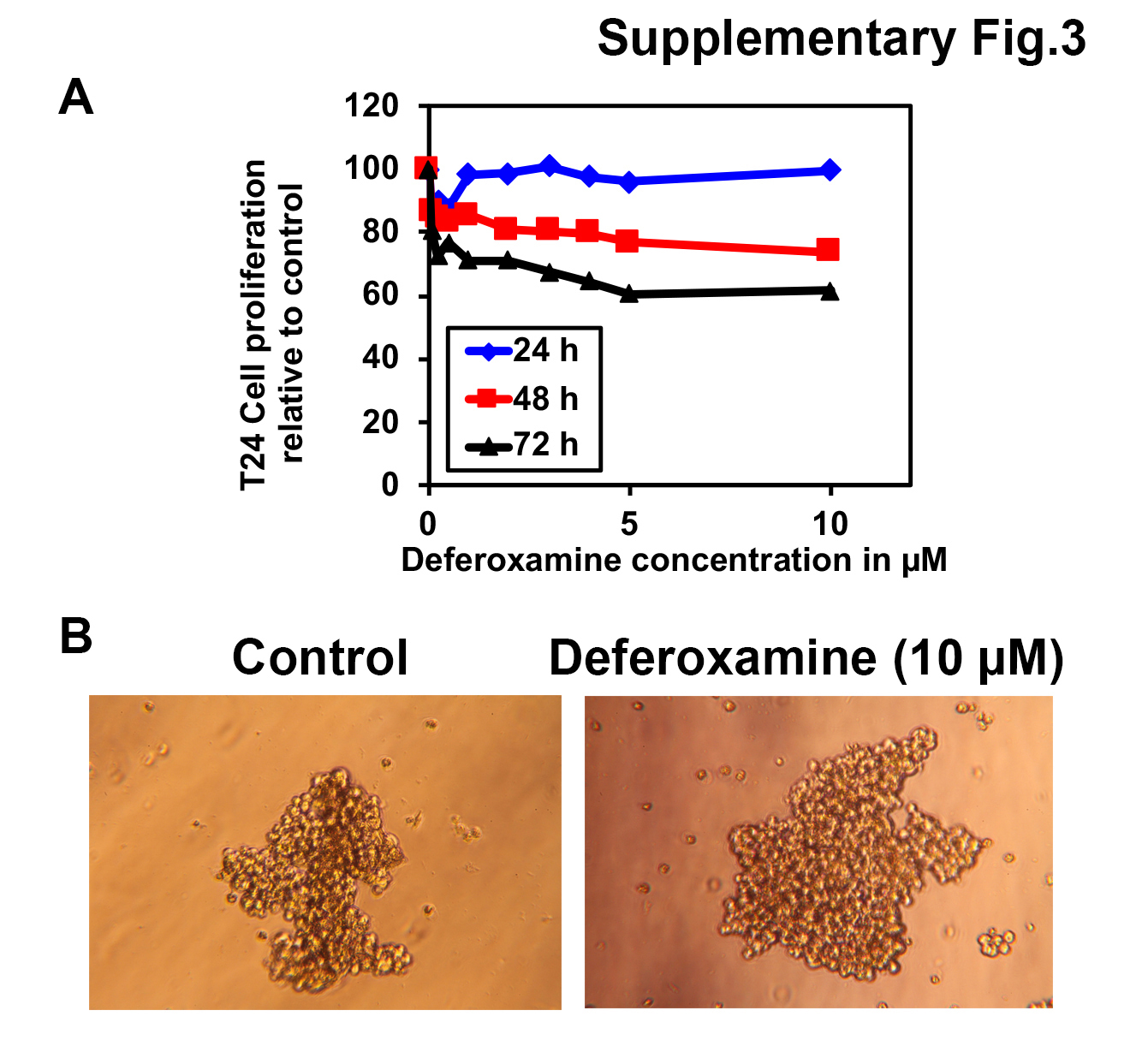

Supplement: Supplementary file 4 — Supplementary Figure 3 [file 41419_2021_3836_MOESM4_ESM.tif]

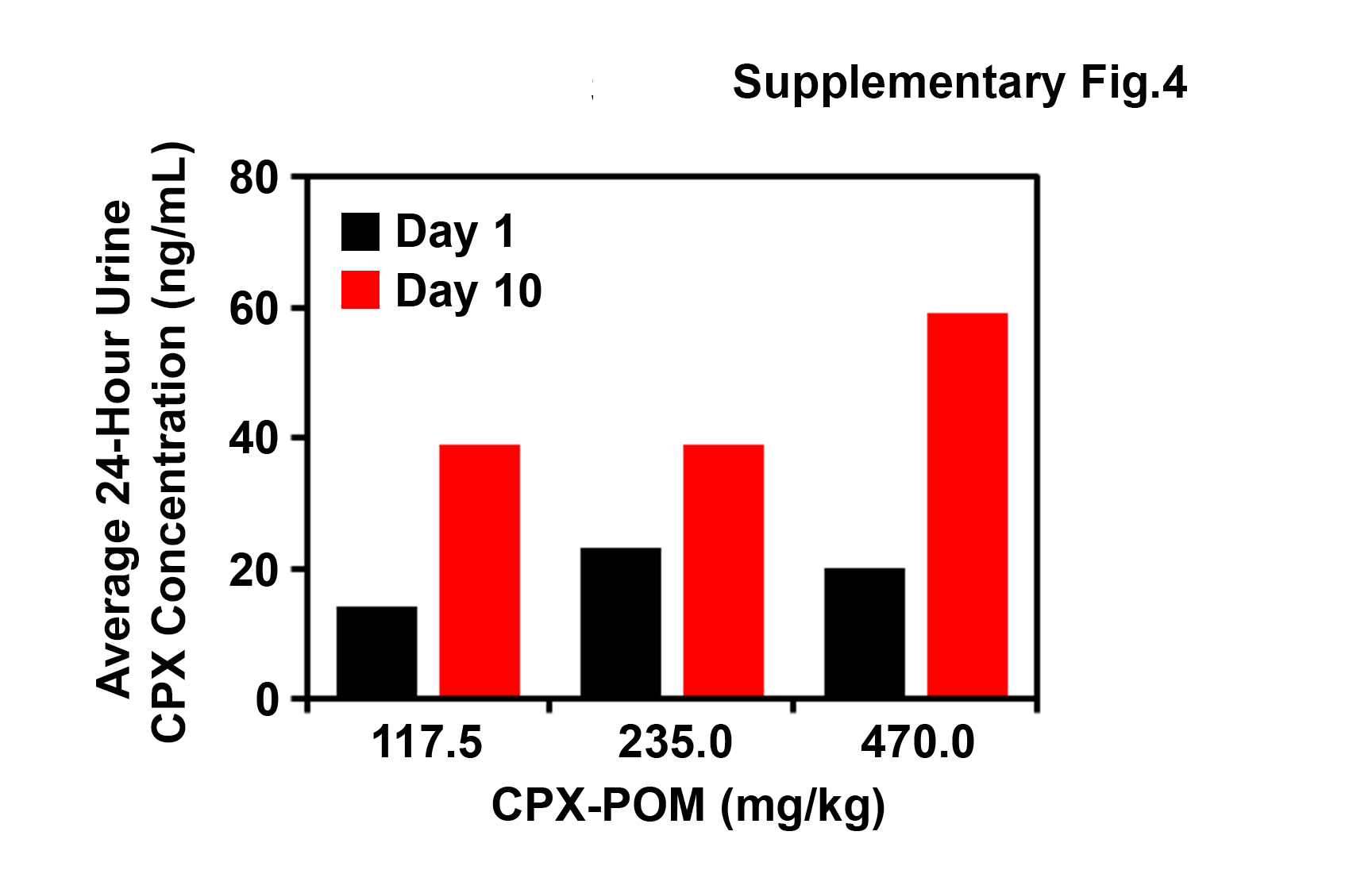

Supplement: Supplementary file 5 — Supplementary Figure 4 [file 41419_2021_3836_MOESM5_ESM.tif]

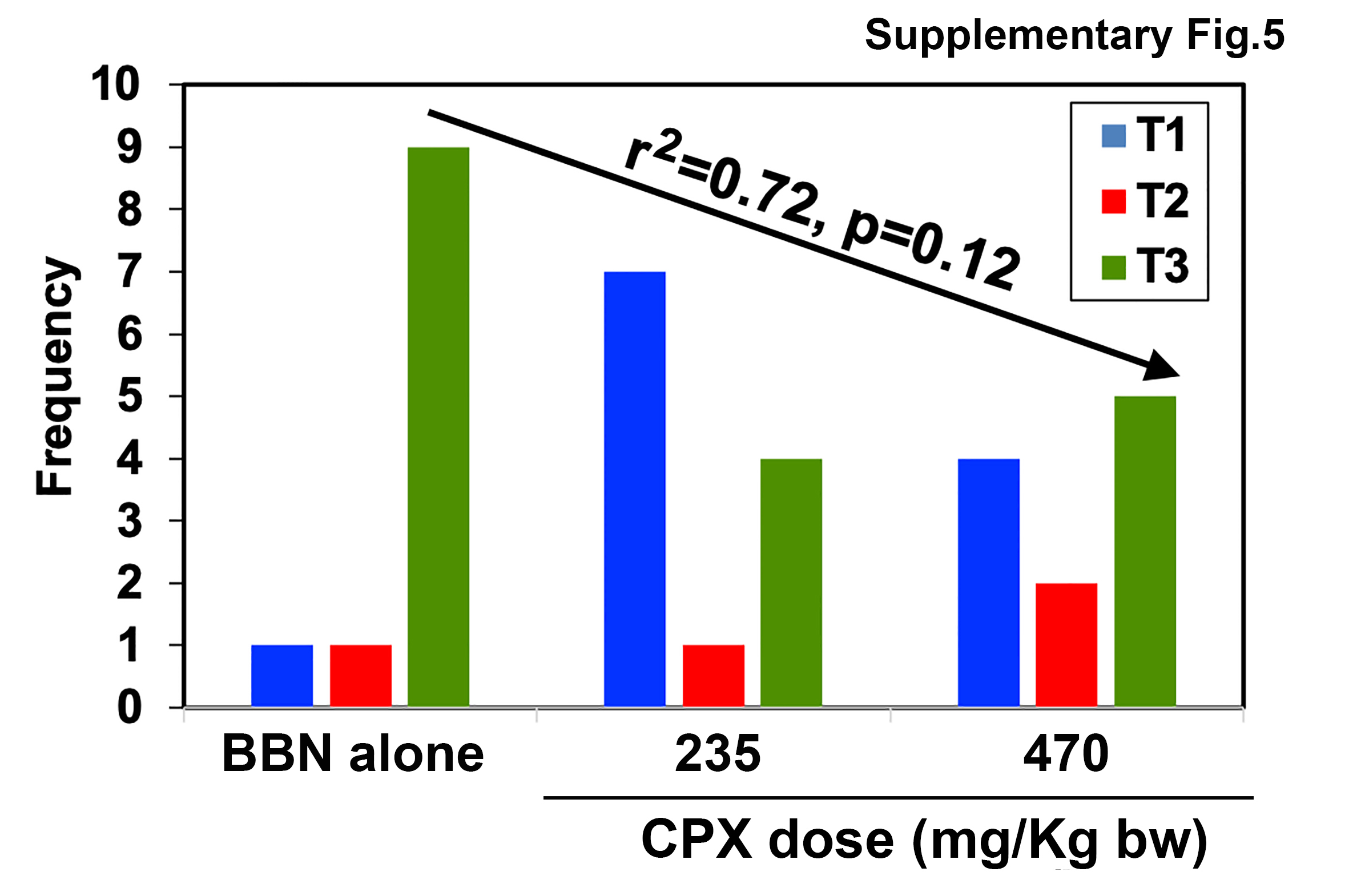

Supplement: Supplementary file 6 — Supplementary Figure 5 [file 41419_2021_3836_MOESM6_ESM.tif]

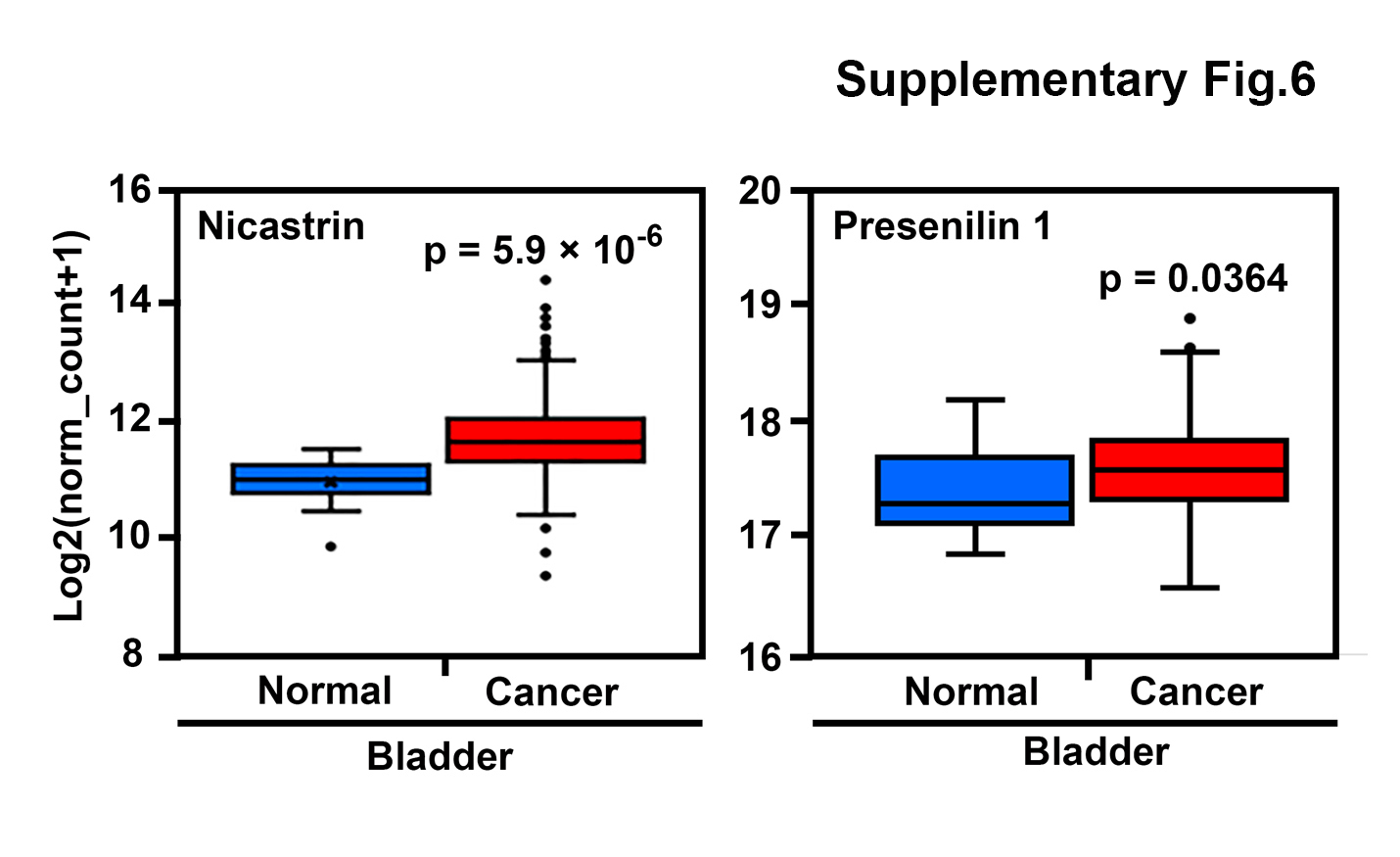

Supplement: Supplementary file 7 — Supplementary Figure 6 [file 41419_2021_3836_MOESM7_ESM.tif]

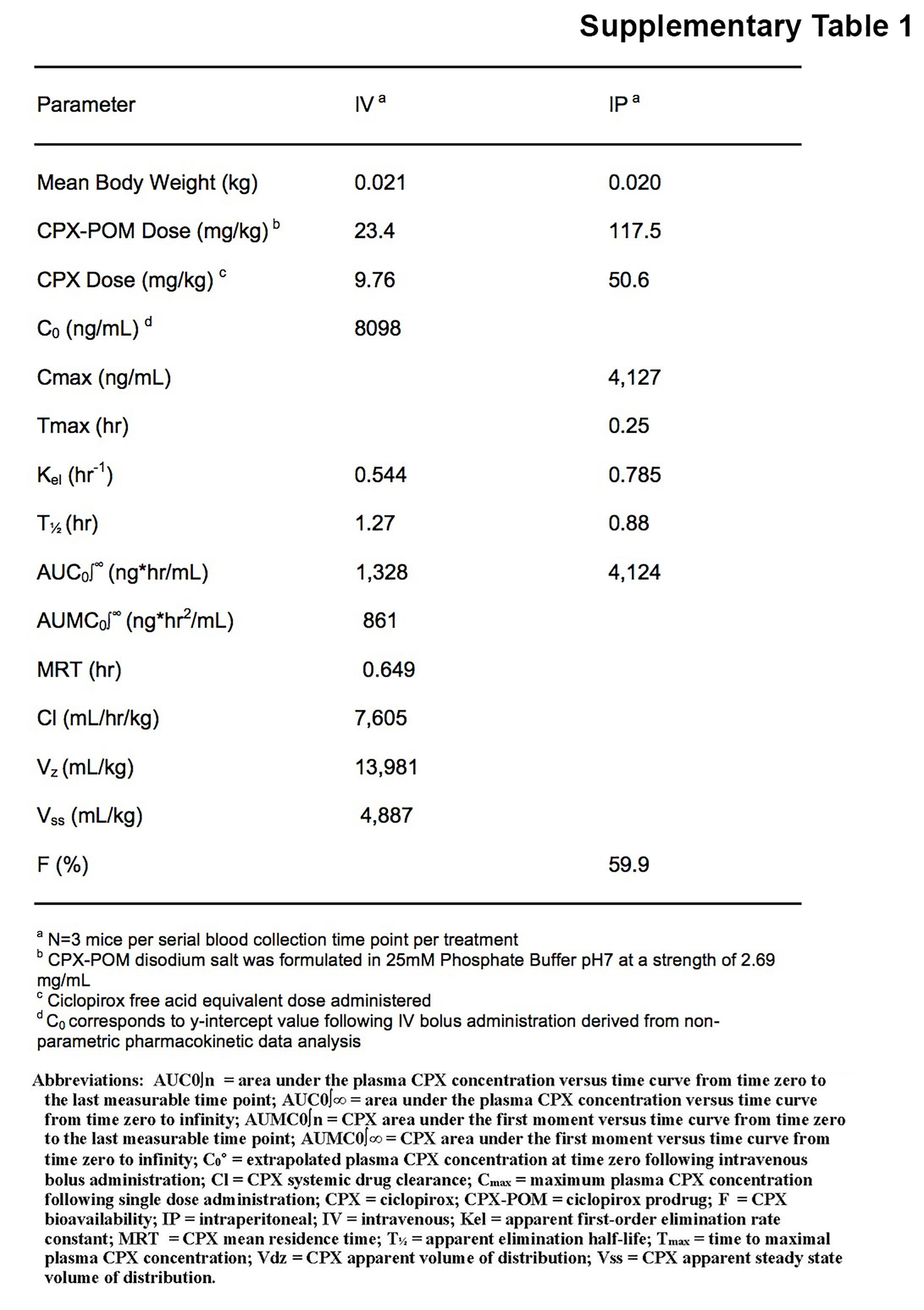

Supplement: Supplementary file 8 — Supplementary Table 1 [file 41419_2021_3836_MOESM8_ESM.tif]
